# A 1,5-Oligosilanylene Dianion as Building Block for Oligosiloxane Containing Cages, Ferrocenophanes, and Cyclic Germylenes and Stannylenes

**DOI:** 10.3390/molecules25061322

**Published:** 2020-03-13

**Authors:** Rainer Zitz, Alexander Pöcheim, Judith Baumgartner, Christoph Marschner

**Affiliations:** Institut für Anorganische Chemie, Technische Universität Graz, Stremayrgasse 9, A-8010 Graz, Austria; Rainer_Zitz@hotmail.com (R.Z.); alexander.poecheim@tugraz.at (A.P.)

**Keywords:** oligosiloxane cage, ferrocenophane, stannylene, distannene, germylene

## Abstract

Starting out from dipotassium 1,5-oligosiloxanylene diide **2**, a 3,7,10-trioxa-octasilabicyclo[3.3.3]undecane was prepared, which represents the third known example of this cage structure type. Reaction of 1,3-dichlorotetramethyldisiloxane with 1,1’-bis[bis(trimethylsilyl)potassiosilyl]ferrocene gave a ferrocenophane with a disiloxane containg bridge. The compound can be further derivatized by conversion into a 1,5-oligosilanyl diide. Reacting 1,5-oligosiloxanylene diide **2** with SnCl_2_ or GeCl_2_·dioxane in the presence of PMe_3_ gave cyclic disilylated tetrylene PMe_3_ adducts. Release of the base-free stannylene led to a dimerization process which gave a bicyclic distannene as the final product. Abstraction of the PMe_3_ from the cyclic disilylated germylene PMe_3_ adduct with B(C_6_F_5_)_3_ caused oxidative addition of the germylene into a para-C-F bond of Me_3_P·B(C_6_F_5_)_3_.

## 1. Introduction

Polysiloxanes (silicones) are an important class of compounds with an almost limitless number of applications. However, despite the huge popularity of these compounds, the vast majority of siloxanes exist as complex mixtures and the synthesis of structurally defined examples has proved to be quite challenging. The reasons for this are strongly associated with the reaction conditions of these acid or base-catalyzed production processes. Siloxanes undergo redistribution reactions of Si-O bonds under these conditions leading to mixtures of different poly- and oligomeric materials together with cyclic compounds.

The recent development of novel synthetic methods, employing neutral conditions has brought the study of oligosiloxanes with exactly defined structures into the focus of several research groups [1,2,3,4,5,6,7,8]. While most of these new methods exploit the activation of hydrosilanes, it has been recognized that the oxidation of oligo- or polysilanes represents another interesting method to obtain oligo- or polysiloxanes retaining the substitution pattern of the starting material [9]. 

**Scheme 1:**
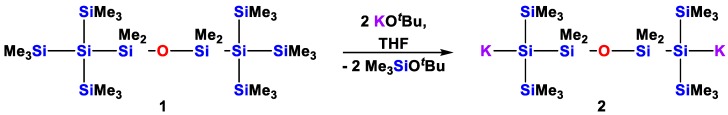
Conversion of 1,3-bis[tris(trimethylsilyl)silyl]tetramethyldisiloxane (**1**) into the 1,5-oligosilanylene diide **2**.

Our own interest in this direction arises from our work on the synthesis of structurally diverse oligosilanes [10]. Utilizing oligosilanyl units as ligands for lanthanides, we recognized the need for additional Lewis basic coordination sites in the backbone of the ligand [11]. We modified the initially used 1,4-tetrasilanylene unit [12,13,14] by introducing an oxygen atom between the two central silicon atoms. The resulting building block is represented by the easily available disiloxane **1**. Its treatment with two equivalents of *^t^*BuOK leads to the respective oligosilanylene diide **2** (Scheme 1) [11], which can be used as a versatile starting material for the preparation of larger molecular architectures containing oligosiloxane substructures [15].

Previously, we reported on the use of compound **2** for the synthesis of a number of small cyclic and acyclic oligosiloxanes as well as its application as a ligand for a number of silylated transition metal complexes [15]. In the current account, we wish to extend these studies further and in particular regarding the use of compound **2** as a ligand for germylene and stannylene units. 

## 2. Results and Discussion

**Scheme 2:**
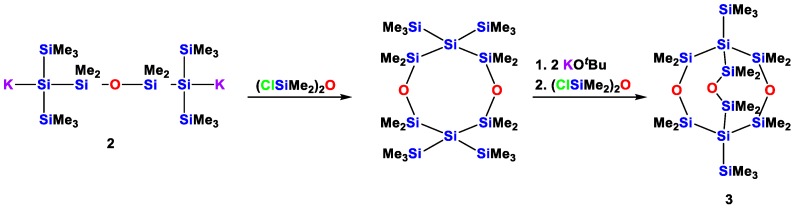
Preparation of a 1,5-dioxacyclooctasilane (**2**), its conversion to the respective 3,7-disilanide, which is further transformed to 1,5-bis(trimethylsilyl)-3,7,10-trioxa-octasilabicyclo[3.3.3]undecane (**3**).

Compound **2** was used to incorporate the bis(isotetrasilanyl)siloxane unit into a number of larger oligosiloxanes [15]. Among these, the tricyclic compound **3** (Scheme 2) is noteworthy as it is a rare example of a tricyclic oligosiloxane with a 3,7,10-trioxa-octasilabicyclo[3.3.3]undecane framework [15]. 

**Chart 1:**
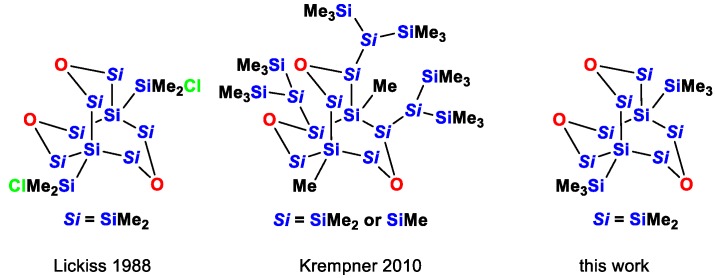
Known examples of compounds containing the 3,7,10-trioxa-octasilabicyclo[3.3.3]undecane framework.

The only other two known instances of this compound class were reported by Lickiss [16] and more recently by Krempner [17] (Chart 1). Lickiss’ compound is very similar to compound **3**, with the only difference being that the trimethylsilyl groups attached to the bridgehead atoms are replaced by dimethylchlorosilyl groups. The compound was formed by careful hydrolysis of (ClMe_2_Si)_4_Si. Although a crystal structure analysis was performed on the compound, the poor quality of the structural solution does not allow an in depth structural discussion [16]. Krempner’s compound is of lower symmetry and was prepared by the reaction of a more complex lithium oligosilanyltriolate [18] with a trichloroisotetrasilane [17]. Furthermore, for this compound, single crystal XRD analysis data are available [17].

**Figure 1:**
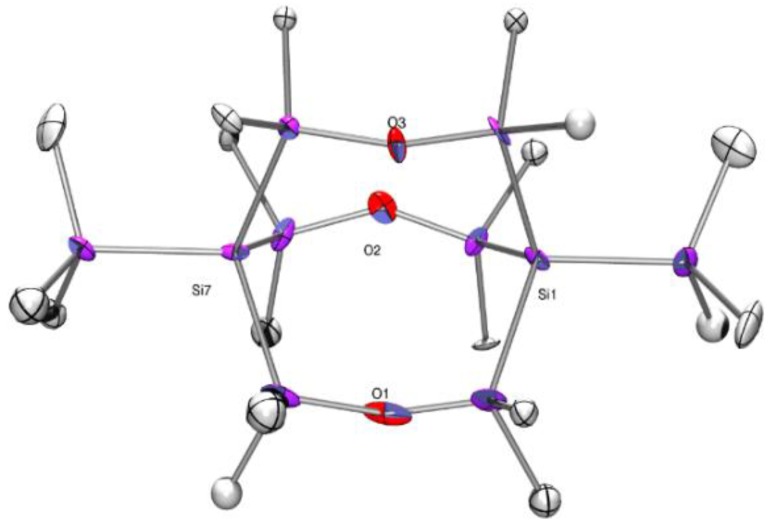
Molecular structure of compound **3** (thermal ellipsoid plot drawn at the 30% probability level). All hydrogen atoms are omitted for clarity (bond lengths in Å, angles in deg). Si(1)-Si(4) 2.3404(18), Si(1)-Si(3) 2.3430(18), Si(1)-Si(2) 2.3486(18), Si(1)-Si(5) 2.3497(18), Si(4)-O(3) 1.641(4), Si(6)-O(3) 1.647(4), Si(8)-O(1) 1.648(4), Si(4)-Si(1)-Si(3) 108.90(7), Si(4)-Si(1)-Si(2) 110.01(7), Si(3)-Si(1)-Si(2) 110.54(7), Si(4)-Si(1)-Si(5) 110.13(7), Si(3)-Si(1)-Si(5) 107.46(7), Si(2)-Si(1)-Si(5) 109.76(7), Si(10)-O(2)-Si(3) 147.9(2), Si(4)-O(3)-Si(6) 148.2(2).

It took us some time to find the correct space group (tetragonal, *P*4_1_) for compound **3** due to its merohedral twinning. The molecular structure features a regular symmetric 3,7,10-trioxa-octasilabicyclo[3.3.3]undecane with trimethylsilyl groups attached to the bridgehead silicon atoms (Figure 1). The Me_3_Si-Si and Si-SiMe_2_O bond distances of 2.35 and 2.34 Å indicate a non strained cage geometry. All Si-O distances are close to 1.64 Å, and the Si-O-Si angles range between 147.9 and 151.7 deg, which is quite typical for this class of compounds. A distance of 4.695 Å was detected between the two bridgehead atoms of compound **3**. This corresponds nicely to a distance of 4.706 Å found for the Lickiss compound. For Krempner’s compound, this distance amounts only to 4.473 Å [17]. The reason for this is likely that the bulky 2-trisilanyl groups in the 2-, 8-, and 9-positions cause a twisting of the 3,7,10-trioxa-octasilabicyclo[3.3.3]undecane cage, which shortens its lengths. For compound **3**, ^29^Si NMR resonances at 10.9, −15.3, and −136.2 ppm for the *Si*Me_3_, *Si*Me_2_, and bridgehead *Si* atoms were found [15], which correlate quite nicely with the 5.5 and −136.4 ppm reported for the analogous groups in Lickiss’ compound [16].

**Scheme 3:**
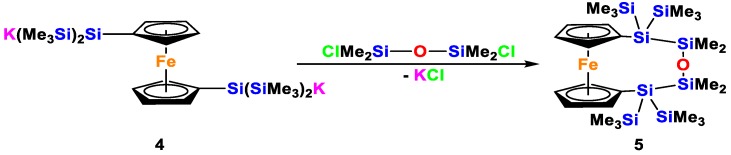
Preparation of the oligosiloxane bridged ferrocene **5** from dianion **4**.

In the course of our previous studies of oligosilanyl dianions, we investigated numerous molecular bridges to connect two tris(trimethylsilyl)silyl groups [19,20,21,22,23,24,25]. In one example, we used a 1,1′-ferrocenylene unit as a spacer. Starting out from tris(trimethylsilyl)silylcyclopentadiene, it is fairly easy to prepare 1,1′-bis[tris(trimethylsilyl)silyl]ferrocene [19]. Reaction of the latter with two equivalents of potassium *tert*-butoxide gives the dianion **4**, which could be used to prepare a number of *ansa*-oligosilanylene bridged ferrocenes [19]. In a similar way, we could react compound **4** with bis(chlorodimethylsilyl)siloxane to obtain compound **5** (Scheme 3). 

**Figure 2:**
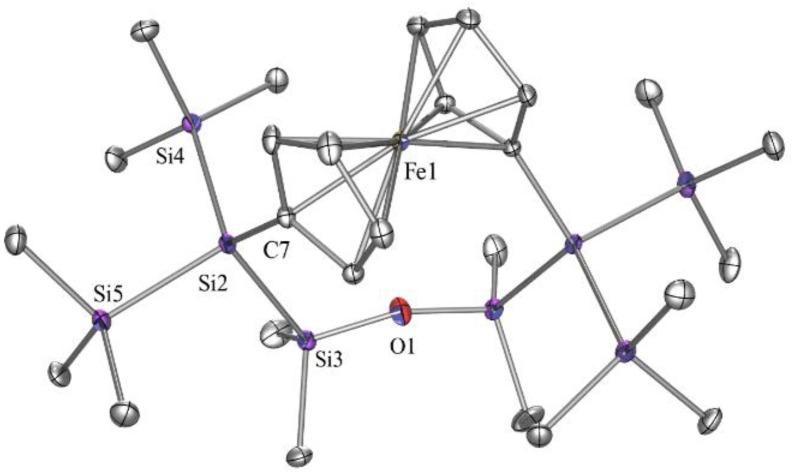
Molecular structure of compound **5** (thermal ellipsoid plot drawn at the 30% probability level). All hydrogen atoms are omitted for clarity (bond lengths in Å, angles in deg). Fe(1)-C(7) 2.080(3), O(1)-Si(3) 1.6250(11), Si(2)-C(7) 1.880(3), Si(2)-Si(3) 2.3474(12), Si(2)-Si(4) 2.3507(12), Si(2)-Si(5) 2.3555(12), C(7)-Si(2)-Si(3) 108.30(10), C(7)-Si(2)-Si(4) 114.63(10),Si(3)-Si(2)-Si(4) 111.57(4), Si(2)-C(7)-Fe(1) 131.76(15).

With the exception of the SiMe_2_O unit, ^1^H, ^13^C, and ^29^Si NMR spectra of compound **5** are similar to that of a related 1,4-tetrasilanylene bridged compound. A ^29^Si NMR chemical shift of 7.0 ppm for the SiMe_2_ group reflects the presence of the comparatively electronegative Cp unit. As there is free rotation around the Cp-Fe bonds, extended lengths of the *ansa*-bridge does not cause steric strain but only increased the separation of the *ipso-*Cp carbon atoms. This can be clearly observed in the solid-state structure of **5** (Figure 2). If we compare the Si-C-C-Si torsional angles of compounds where the *ansa-*bridge consists of either a 1,2-disilanylene [19] or a 1,4-tetrasilanylene [19] unit with that of compound **5**, we observe an increase in the value from ca. 20 deg to 55 deg and 70 deg for compound **5**. The Si-O-Si angle is 156.12 deg, which is a typical value for this unit in acyclic or larger ring systems [15]. 

**Scheme 4:**
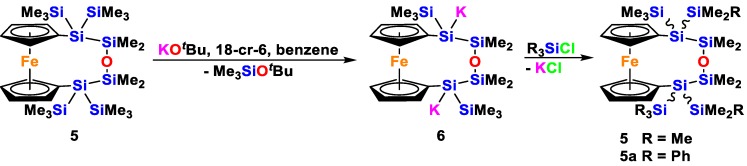
Reaction of oligosiloxane bridged ferrocene **5** with two equivalents of *^t^*BuOK to form the expected dianionic compound **6,** which was derivatized either with Me_3_SiCl or PhMe_2_SiCl.

In an attempt to generate an oligosilanyl dianion from **5**, we reacted it with two equivalents of KO*^t^*Bu. Despite a seemingly very fast reaction, we were not able to detect the supposedly formed dianionic compound **6** NMR spectroscopically. In addition to formation of Me_3_SiO*^t^*Bu, the spectra (^1^H, ^13^C and ^29^Si) only showed the formation of rather broad peaks in the trimethylsilyl region. Nevertheless, within minutes of reaction, a deep red precipitate formed, which reacts upon addition of trimethylchlorosilane to give compound **5**, and when treated with phenyldimethylchlorosilane gives a mixture of two diasteromers **5a** in an approximate 1:1 ratio (Scheme 4). We observed such a behavior before [19,20], which is caused by the configurational lability of silanides.

**Scheme 5:**
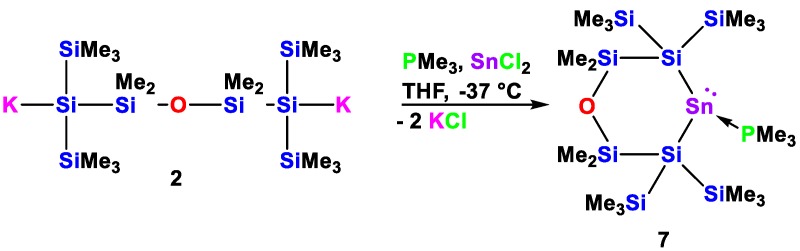
Reaction of 1,5-oligosilanylene diide **2** with SnCl_2_·PMe_3_ to the cyclic silylated stannylene PMe_3_ adduct **7**.

**Scheme 6:**
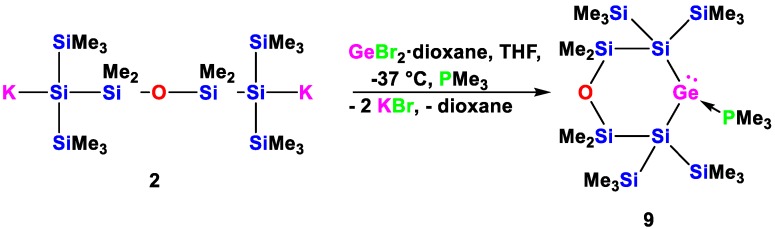
Reaction of 1,5-oligosilanylene diide **2** with GeCl_2_ in the presence of PMe_3_ to the cyclic silylated germylene PMe_3_ adduct **9**.

For the last part of this study, we decided to react disilanide **2** with GeCl_2_·dioxane and SnCl_2_ in the presence of PMe_3_ to test the formation of germylene and stannylene phosphane adducts (Scheme 5 and Scheme 6). Related reactions of two equivalents of potassium tris(trimethylsilyl)silanide with either ECl_2_·NHC (E = Ge, Sn; NHC = 1,3-diisopropyl-4,5-dimethyl-imidazol-2-ylidene (^Me^IiPr)) [26] or ECl_2_·PR_3_ (E = Ge, Sn; PR_3_ = PMe_3_, PEt_3_) [27,28,29] and of 1,3- and 1,4-oligosilanyldiides with ECl_2_·PR_3_ (E = Ge, Sn; PR_3_ = PMe_3_, PEt_3_) [30,31,32,33] gave Lewis base adducts of either acyclic or four- and five-membered cyclic disilylated germylenes and stannylenes. 

Not unexpectedly, a clean formation of stannylene PMe_3_ adduct **7** was observed in the reaction of compound **2** with SnCl_2_·PMe_3_ (Scheme 5) at low temperature.

^1^H, ^13^C, and ^29^Si NMR spectra of compound **7** show two signal sets for the methyl and trimethylsilyl groups on the different sides of the ring, indicating configurational stability of the tin atom with a stereochemically active lone pair. The same was observed before at the stannacyclopentasilane analog with PEt_3_ serving as base [30]. The ^119^Sn NMR resonance of compound **7** was found at −167.9 ppm, which is somewhat downfield from the −224.4 ppm observed for the related stannacyclopentasilane analog [30]. For the two examples of bis[tris(trimethylsilyl)silyl]stannylene phosphane adducts with PEt_3_ [28] and PMe_3_ [33], the respective ^119^Sn NMR resonances were detected at −113.3 and −89.9 ppm. When these values are correlated with the Si-Sn-Si angles observed in the solid state, it becomes evident that the chemical shift goes downfield with increasing Si-Sn-Si angle, which correlates with a diminished singlet–triplet gap. Although the ^31^P NMR resonance of compound **7** (−62.5 ppm) is very close to that of free PMe_3_, coupling to ^117^Sn and ^119^Sn can be observed very well. 

**Figure 3:**
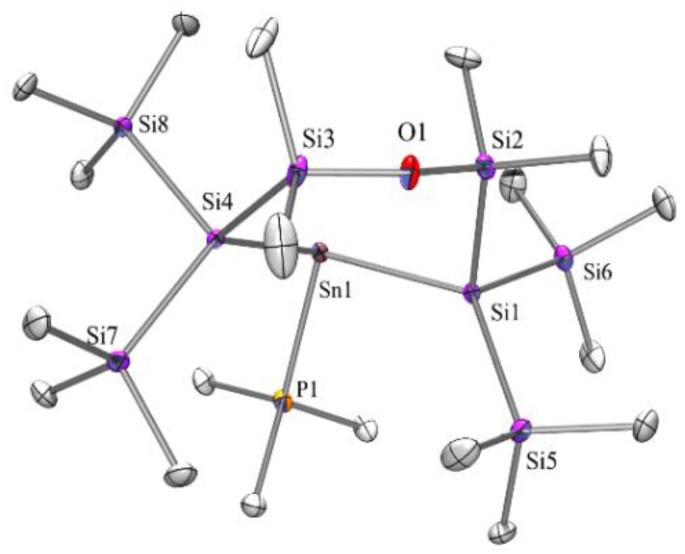
Molecular structure of compound **7** (thermal ellipsoid plot drawn at the 30% probability level). All hydrogen atoms are omitted for clarity (bond lengths in Å, angles in deg). C(1)-P(1) 1.814(3), C(4)-Si(2) 1.866(3), O(1)-Si(3) 1.6378(19), O(1)-Si(2) 1.642(2), P(1)-Sn(1) 2.6059(8), Si(1)-Si(2) 2.3397(11), Si(1)-Sn(1) 2.6464(8), Si(4)-Sn(1) 2.6662(8), Si(3)-O(1)-Si(2) 145.51(13), Si(2)-Si(1)-Sn(1) 96.56(3), Si(3)-Si(4)-Sn(1) 109.20(3), P(1)-Sn(1)-Si(1) 99.58(2), P(1)-Sn(1)-Si(4) 104.33(2), Si(1)-Sn(1)-Si(4) 103.35(2).

The single crystal XRD analysis of compound **7** (Figure 3) exhibits Sn-Si distances of 2.6465(8) and 2.6662(8) Å, a Sn-P distance of 2.6059(9) Å, and a Si-Sn-Si angle of 103.35(2) deg. While the distances in the stannacyclopentasilane analog are very similar, the Si-Sn-Si angle of that compound is smaller (98.17(9) deg). For the acyclic compounds [(Me_3_Si)_3_Si]_2_Sn·PR_3_ (R = Et. Me) [28,33], the Sn-Si distances range between 2.686(3) and 2.717(1) Å and the Si-Sn-Si angles increase to 114.21(4) (PEt_3_) and 117.53(6) (PMe_3_) deg.

**Scheme 7:**
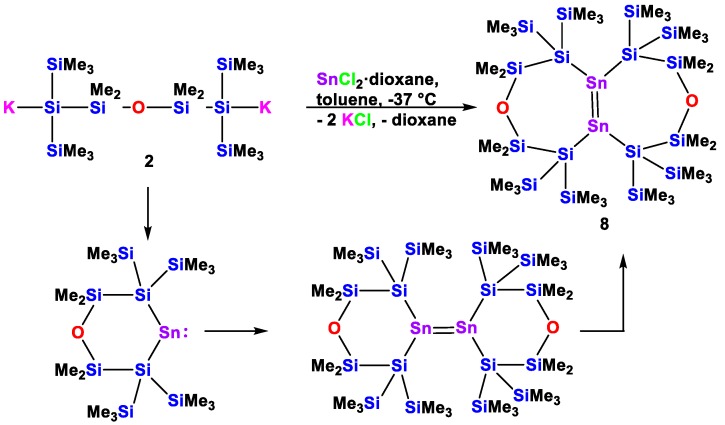
Reaction of 1,5-oligosilanylene diide **2** with SnCl_2_ in the absence of a donating Lewis base to the bicyclic tetrasilylated distannene **8**.

When the reaction of compound **2** with SnCl_2_ was repeated in the absence of a strongly donating agent (PR_3_, NHC), the formation of a bicyclic distannene (**8**) was observed. In addition, this reaction course follows what we previously observed for the reactions of dipotassio α,α,ω,ω-tetrakis(trimethylsilyl)oligosilanyl-α,ω-diiides with SnCl_2_ [30,33]. For this reason we suppose that compound **8** forms by dimerization of an initially formed six-membered cyclic disilylated stannylene to an exocyclic distannene, which finally rearranges via two 1,2-silyl shifts to compound **8** (Scheme 7). For the bicyclic distannene with two annulated six-membered rings, support for this reaction mechanism was obtained by a computational study [30].

**Chart 2:**
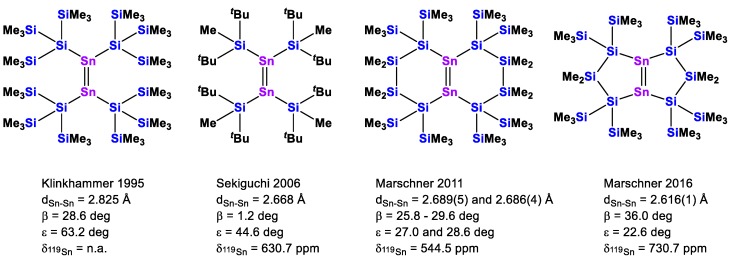
Known tetrasilylated distannenes with key structural values of Sn-Sn bond distance, bending angle β, twisting angle ε, and ^119^Sn NMR chemical shift value.

Compound **8** adds to a growing number of known tetrasilylated distannenes. Starting with Klinkhammer’s bis[tris(trimethylsilyl)silyl]stannylene, which exists as a distannene in the solid state [34], followed by Sekiguchi’s tetrakis(di-*tert*-butylmethylsilyl)distannene [35], our group added two further examples of bicyclic distannenes [30,33] (Chart 2). 

**Figure 4:**
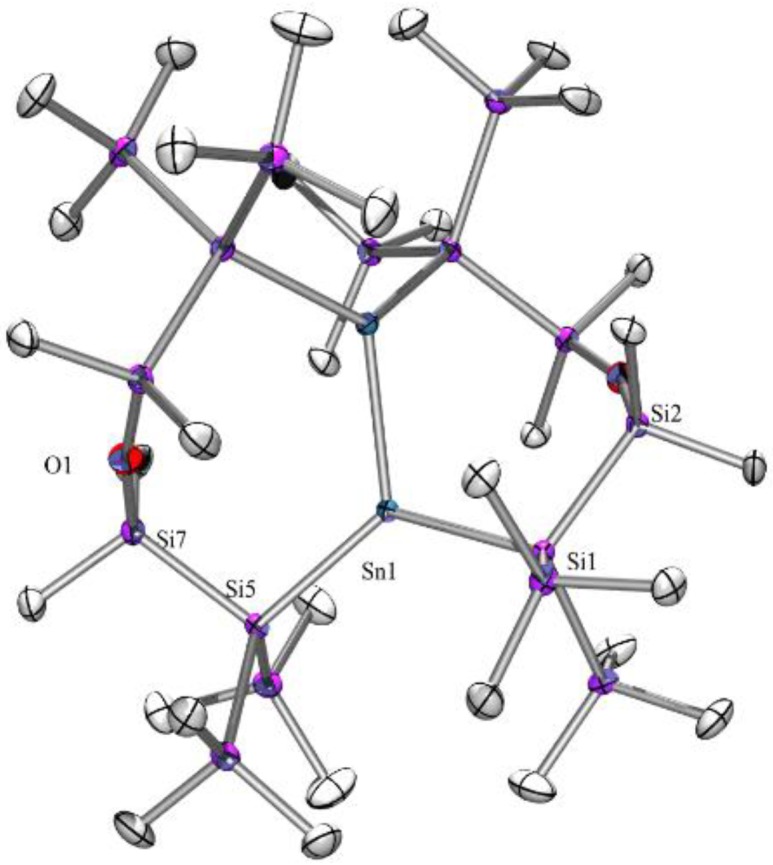
Molecular structure of compound **8** (thermal ellipsoid plot drawn at the 30% probability level). All hydrogen atoms are omitted for clarity (bond lengths in Å, angles in deg). Sn(1)-Si(1) 2.6131(8), Sn(1)-Si(5) 2.6179(8), Sn(1)-Sn(1A) 2.7409(9), Si(1)-Si(2) 2.3557(10), Si(7)-O(1) 1.6302(8), Si(7)-C(14) 1.866(3), Si(1)-Sn(1)-Si(5) 122.90(3), Si(1)-Sn(1)-Sn(1A) 105.336(17), Si(5)-Sn(1)-Sn(1A) 122.683(18), Si(7)-O(1)-Si(7A) 168.10(19).

The ^119^Sn NMR chemical shift of compound **8** (636.9 ppm) is very close to Sekiguchi’s compound (*^t^*Bu_2_MeSi)_2_Sn = Sn(SiMe*^t^*Bu_2_)_2_ (see Chart 2). In the crystal structure of compound **8** (Figure 4), the bending angle is 31.1 deg, but its twisting angle is rather large. However, while (*^t^*Bu_2_MeSi)_2_Sn = Sn(SiMe*^t^*Bu_2_)_2_ is a rather symmetric molecule with respect to the twisting of the stannylene units, the situation in compound **8** is somewhat more complicated. The two rings of the bicyclic molecules exhibit different conformations, with one ring showing a small Si-Sn-Sn-Si torsional angle of 17.0 deg, while for the other ring, the angle measured was 81.6 deg. The Sn=Sn double bond distance of 2.7409(9) Å, as observed for compound **8,** is the longest of the three examples of bicyclic disilylated stannanes (Chart 2) [30,33]. It seems that increasing ring size and likely the associated increased ring strain cause a diminished interaction of the two stannylene units. This does not, however, correlate with the respective ^119^Sn NMR chemical shifts.

Reacting oligosilanyldiide **2** with GeCl_2_·dioxane in the presence of PMe_3_ gave the germylene PMe_3_ adduct (Scheme 6) in a clean reaction. Again, this is in agreement with similar reactions to acyclic [29,36] and cyclic [32,37] disilylated germylenes we have reported before. The obtained compound **9** features the expected six-membered ring with a germylene being coordinated by a PMe_3_ molecule as base. 

**Figure 5:**
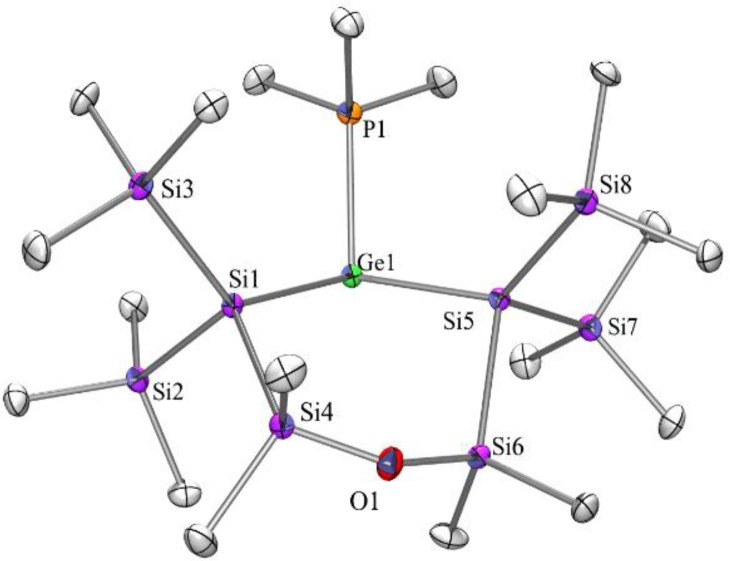
Molecular structure of compound **9** (thermal ellipsoid plot drawn at the 30% probability level). All hydrogen atoms are omitted for clarity (bond lengths in Å, angles in deg). Ge(1)-P(1) 2.3570(13), Ge(1)-Si(5) 2.4463(13), Ge(1)-Si(1) 2.4664(13), O(1)-Si(4) 1.625(3), O(1)-Si(6) 1.642(3), P(1)-C(1) 1.818(5), Si(1)-Si(4) 2.3656(17), Si(2)-C(9) 1.879(5), Si(5)-Si(6) 2.3537(17), P(1)-Ge(1)-Si(5) 100.20(5), P(1)-Ge(1)-Si(1) 101.87(4), Si(5)-Ge(1)-Si(1) 107.88(5), Si(4)-O(1)-Si(6) 148.4(2), Si(4)-Si(1)-Ge(1) 110.79(6).

^1^H, ^13^C, and ^29^Si NMR spectra of compound **9** again show two signal sets for the methyl and trimethylsilyl groups on the different sides of the ring, thus indicating configurational stability of the germylene unit. However, the trimethylsilyl groups show clear signs of signal broadening, suggesting that at rt conditions, coalescence has already started. For the related germacyclopentasilane analog with PEt_3_ serving as base [37], the signals at rt are much broader, indicating a higher degree of inversion. However, it is not clear whether the increased configurational stability of compound **9** is based on the different ligand and different Si-Ge-Si angle or the stronger interaction between the germylene and PMe_3_ compared to PEt_3_. The Si-Ge-Si angle of compound **9** in the solid state (Figure 5) of 107.88(4) deg is larger than the respective angle of compound **7** (103.35(2) deg), which can be explained by the shorter Si-Ge bonds. The same angle in the five-membered ring examples [37,38] is also smaller (101.3(3) and 102.70(1) deg). It is known that the geometry of the tetrylene has a decisive influence on the singlet–triplet gap [39], so it can be assumed that the respective free germylene of compound **9** exhibits a higher reactivity than the examples with smaller Si-Ge-Si angles.

**Scheme 8:**
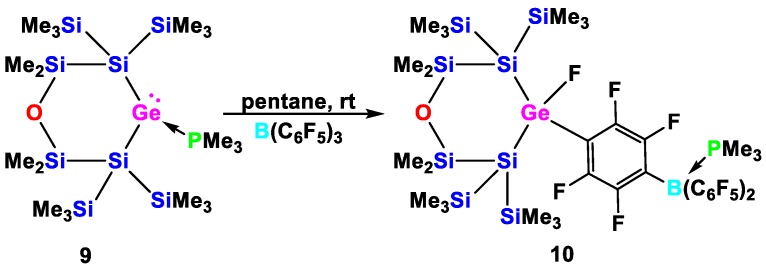
Reaction of germylene adduct **9** with B(C_6_F_5_)_3_ to abstract the PMe_3_ to release the free germylene, which in a subsequent step inserts into a para-C-F bond of B(C_6_F_5_)_3_·PMe_3_ to give compound **10**.

In previous studies, we found that the addition of strong donor molecules like phosphanes or N-heterocyclic carbenes (NHCs) is essential to achieve a clean germylene formation in the reaction of silanides with GeCl_2_. In order to get access to the free germylene from its phosphane adduct, treatment with a fairly strong Lewis acid proved to be a good strategy. The Lewis acid would abstract the donor and release the germylene. Massey’s borane B(C_6_F_5_)_3_ [40] was found to be a particularly good reagent in this respect. When compound **9** was treated with B(C_6_F_5_)_3_, we expected PMe_3_ abstraction, followed by the formation of the free germylene, which could dimerize in a similar way as shown above for the analogous stannnylene. Alternatively, we also observed several instances where the germylene undergoes a 1,2-silyl shift to the germanium atom, thus forming a silagermene, which might undergo a 2 + 2 cycloaddition reaction in a subsequent step. However, in the current case, our expectations were not met and another reaction pathway was observed. While the PMe_3_ abstraction seemingly occurred as expected, the free germylene reacted by insertion into a p-C-F bond of (C_6_F_5_)_3_B·PMe_3_ (Scheme 8). This is quite unexpected as we have never before observed activation of C-F bonds in related reactions. Nevertheless, the reaction is not completely without precedence. Roesky and co-workers observed C-F activation of heteroleptic chloro- and amino-silylenes (LSiX, X = Cl, NR_2,_ L = PhC(NtBu)_2_) stabilized by a benzamidinato ligand with *tert*-butyl substituents on the nitrogen atoms [41,42]. Related reactivity was even observed with germylenes and stannylenes [43]. One particular aspect of this chemistry is that there is some completion between oxidative addition of C-F bonds and fluoride metathesis to products, where the divalent character of the tetrylene is retained and only the substituent is exchanged for fluoride. This chemistry has also been studied theoretically [44]. 

The fact that this oxidative addition occurs with the free germylene of compound **9** but was not observed with the previously studied cyclic disilylated germylenes suggests a higher reactivity, which is likely caused by the larger Si-Ge-Si bond angle which favors a smaller singlet–triplet gap [39]. 

NMR spectra of compound **10** feature a combination of the cyclooligosilyloxygermanium part similar to in compound **9** with signals similar to that of Me_3_P·B(C_6_F_5_)_3_ [45] and B(*p*-C_6_F_4_H)_3_ [46]. The ^1^H, ^13^C, and ^29^Si spectra mainly represent the cyclooligosilyloxygermanium fragment. With two types of methyl and trimethylsilyl resonances indicating ring-side differentiation. The borate adduct part of compound **10** is mainly represented in the ^19^F and ^31^P NMR spectra. The resonance for the Ge-F unit in the ^19^F NMR spectrum was detected at −209.2 ppm. While no similar germanium fluoride is known, the chemical shift of the related silane [(Me_3_Si)_2_MeSi]_2_PhSiF at −193.5 ppm [47] supports the assignment.

**Figure 6:**
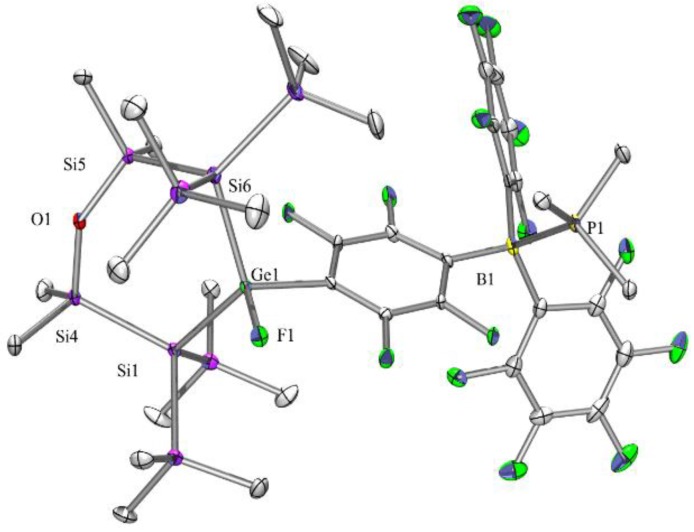
Molecular structure of compound **10** (thermal ellipsoid plot drawn at the 30% probability level). All hydrogen atoms are omitted for clarity (bond lengths in Å, angles in deg). Ge(1)-F(1) 1.769(2), Ge(1)-C(17) 1.969(4), Ge(1)-Si(6) 2.3849(13), Ge(1)-Si(1) 2.3966(12), O(1)-Si(5) 1.654(3), O(1)-Si(4) 1.654(3), P(1)-C(35) 1.805(5), P(1)-B(1) 2.055(5), Si(1)-Si(4) 2.3775(17), Si(2)-C(1) 1.860(5), Si(5)-Si(6) 2.3447(17), B(1)-C(29) 1.632(7), F(10)-C(28) 1.347(6), F(1)-Ge(1)-Si(6) 105.08(9), F(1)-Ge(1)-Si(1) 104.92(9), Si(6)-Ge(1)-Si(1) 119.41(4), Si(5)-O(1)-Si(4) 139.1(2), Si(4)-Si(1)-Ge(1) 107.01(6), Si(5)-Si(6)-Ge(1) 101.08(5).

In the solid-state structure of compound **10** (Figure 6), the rather short Si-Ge distances of 2.385(1) and 2.397(1) Å reflect the fact that bonds to tetravalent germanium are shorter. For the same bonds in compound **9** (2.446(2) and 2.466(1) Å), significantly longer distances were observed.

## 3. Experimental Section

### 3.1. General Remarks

All reactions involving air-sensitive compounds were carried out under an atmosphere of dry nitrogen or argon using either Schlenk techniques or a glove box. Solvents were dried using a column-based solvent purification system [48]. 1,3-Bis[tris(trimethylsilyl)silyl]-1,1,3,3-tetramethyldisiloxane (**1**) [11], **2** [11], **3** [15], and **4** [19] were prepared according to previously published procedures. All other chemicals were obtained from different suppliers and used without further purification.

^1^H (300 MHz), ^13^C (75.4 MHz), ^19^F ((282.2 MHz), ^29^Si (59.3 MHz), ^31^P (124.4 MHz), and ^119^Sn (111.8 MHz) NMR spectra were recorded on a Varian INOVA 300 spectrometer and are referenced to tetramethylsilane (TMS) for ^1^H, ^13^C, and ^29^Si, to CFCl_3_ for ^19^F, to 85% H_3_PO_4_ for ^31^P and to Me_4_Sn for ^119^Sn. If not otherwise noted, the solvent used was C_6_D_6_, and samples were measured at rt. In case of reaction samples, a D_2_O capillary was used to provide an external lock frequency signal. To compensate for the low isotopic abundance of ^29^Si, the INEPT pulse sequence [49,50] was used for the amplification of the signal for some of the spectra. 

Elementary analyses were carried out using a Heraeus VARIO ELEMENTAR instrument. For a number of compounds, no good elemental analysis values could be obtained, which is a typical problem for these compounds, caused primarily by silicon carbide formation during the combustion process. Multinuclear NMR spectra (^1^H, ^13^C, ^29^Si) of these compounds are presented in the supplementary materials (Figures S1–S22) as proof of purity.

### 3.2. X-ray Structure Determination

For X-ray structure analyses, the crystals were mounted onto the tip of glass fibers, and data collection was performed with a BRUKER-AXS SMART APEX CCD diffractometer using graphite-monochromated Mo K_α_ radiation (0.71073 Å). The data were reduced to F^2^_o_ and corrected for absorption effects with SAINT [51] and SADABS [52,53], respectively. The structures were solved by direct methods and refined using the full-matrix least-squares method (SHELXL97) [54]. If not otherwise noted, all non-hydrogen atoms were refined with anisotropic displacement parameters, and all hydrogen atoms were located in calculated positions to correspond to standard bond lengths and angles. Crystallographic data (excluding structure factors) for the structures of compounds **3**, **5**, **7**, **8**, **9**, and **10** reported in this paper have been deposited with the Cambridge Crystallographic Data Center as supplementary publication (Table S1) No. CCDC-1982093 (**3**), 1982094 (**5**), 1982096 (**7**), 1982098 (**8**), 1982097 (**9**), and 1982095 (**10**). Data can be obtained free of charge at: http://www.ccdc.cam.ac.uk/products/csd/request/. Figures of solid-state molecular structures were generated using Ortep-3 as implemented in WINGX [55] and rendered using POV-Ray 3.6 [56].

### 3.3. Synthesis

#### 3.3.1. 1,1′-ansa-[2,2,4,4-Tetramethyl-1,1,5,5-tetrakis(trimethylsilyl)-3-oxa-1,2,4,5-tetrasila-1,5-diyl]ferrocene (**5**)

1,1′-Bis[tris(trimethylsilyl)silyl]ferrocene (588 mg, 0.87 mmol) and KO*^t^*Bu (200 mg, 1.78 mmol) were dissolved in THF (5 mL) and after 20 min a bright red color was observed. After 22 h, the solution was added dropwise within 2 min to a solution of 1,3-dichloro-1,1,3,3-tetramethyldisiloxane (182 mg, 0.90 mmol) in toluene (5 mL), and the reaction mixture was stirred for 6 h. Volatiles were removed under reduced pressure, pentane was added (3 × 3 mL), centrifuged, and filtrated. Orange crystals of compound **5** (560 mg, 97%) were obtained from pentane at −62 °C. NMR (δ in ppm): ^1^H: 4.23 (m, 4H), 4.11 (m, 4H), 0.51 (s, 12H), 0.26 (s, 36H).^13^C: 75.9, 70.7, 66.7, 7.2, 1.3. ^29^Si: 7.0 (s, SiMe_2_), −13.9 (SiMe_3_), −84.0 (Siq).

#### 3.3.2. 1,1’-ansa-[1,5-Dipotassio-2,2,4,4-tetramethyl-1,5-bis(trimethylsilyl)-3-oxa-1,2,4,5-tetrasila-1,5-diyl]ferrocene (**6**)

A solution of compound **5** (100 mg, 0.15 mmol) in benzene (1 mL) was added dropwise to a solution of KO*^t^*Bu (35 mg, 0.31 mmol) and 18-cr-6 (86 mg, 0.33 mmol) in benzene (1 mL). Immediately, the color changed from orange to bright red. After 1 min, a luminous bright orange precipitate formed. After 5 h at rt, the supernatant was decanted and the precipitate was dissolved in THF and subjected to NMR-analysis, which showed only a single signal at −11.1 ppm along with trace amounts of silyl ether in the ^29^Si NMR spectrum, hence not giving clear evidence for the formation of compound **6**. Therefore, the solution was treated with chlorodimethylphenylsilane (89 mg, 0.52 mmol) in THF (1 mL). After 2.5 h at rt, the reaction mixture was subjected to NMR-analysis showing a diastereotopic mixture of compound **5a**. NMR (δ in ppm): ^29^Si: 7.5/7.4 (s, SiMe_2_), −13.4/−13.5 (SiMe_3_), −17.7/−18.1 (SiPhMe_2_), −83.2/−83.4 (Siq).

#### 3.3.3. 2-Stanna-5-oxa-1,1,3,3-tetrakis(trimethylsilyl)tetramethylcyclohexasilan-2-ylidene·PMe_3_ (**7**)

A mixture of SnCl_2_ (30 mg, 0.16 mmol) and PMe_3_ (18 mg, 0.24 mmol) was dissolved in THF (2 mL), stirred for 10 min at rt, and cooled to −37 °C. To this, a solution of compound **2** (0.16 mmol) in THF (1 mL) was added dropwise resulting in an red–brown solution. After stirring for 1 h, the solvent was removed in a vacuum and the residue was extracted with pentane (3 × 2 mL). Filtration over celite was followed by concentration of the volume. Crystallization at −37 °C provided yellow crystalline blocks of compound **7** (47 mg, 44%). Mp.: 154–156 °C. NMR (δ in ppm): ^1^H: 1.10 (d, 9H, ^2^*J*_H-P_ = 8.6 Hz, PMe_3_), 0.57 (bs, 6H, OSiMe_2_), 0.51 (bs, 6H, OSiMe_2_), 0.45 (bs, 18H, SiMe_3_), 0.39 (bs, 18H, SiMe_3_). ^13^C: 18.2 (d, ^1^*J*_C-P_ = 16.4 Hz, PMe_3_), 8.7 (OSiMe_2_), 8.4 (OSiMe_2_), 5.0 (SiMe_3_), 3.5 (SiMe_3_). ^29^Si: 12.9 (d, ^3^*J*_Si-P_ = 9 Hz, OSiMe_2_), −6.1 (d, ^3^*J*_Si-P_ = 14 Hz, SiMe_3_), −9.9 (bs, SiMe_3_), −142.4 (d, ^3^*J*_Si-P_ = 16 Hz, d, ^1^*J*_Si117-119Sn_ = 569/575 Hz, SiSn). ^31^P: −62.5 (^1^*J*_P-Sn117/119Sn_ = 1991/2084 Hz, SnPMe_3_). ^119^Sn: −167.9 (^1^*J*_P-119Sn_ = 2084 Hz, SnPMe_3_).

#### 3.3.4. 1,7-Distanna-2,2,6,6,8,8,12,12-octakis(trimethylsilyl)-4,10-dioxabicyclo[5.5.0]-dodecasil-1,7-ene (**8**)

Compound **8** was obtained in an analogous way as described below for compound **9** using SnCl_2_ (60 mg, 0.32 mmol), ^t^BuOK (74 mg, 0.78 mmol), and **2** (200mg, 0.16 mmol). Green crystals (which turn to orange/red under polarized light) of compound **8** (47 mg, 64%) were formed in a toluene solution at −37 °C. Mp.: 167–170 °C. NMR (δ in ppm): ^1^H: 0.59 (s, 24H, OSiMe_2_), 0.44 (s, 72H, SiMe_3_). ^13^C: 8.3 (OSiMe_2_), 4.1 (SiMe_3_).^29^Si: 13.75 (OSiMe_2_), −9.0 (SiMe_3_), −84.9 (SiSn). ^119^Sn: 636.9.

#### 3.3.5. 2-Germa-5-oxa-1,1,3,3-tetrakis(trimethylsilyl)tetramethylcyclohexasilan-2-ylidene·PMe_3_ (**9**)

A mixture of GeBr_2_·dioxane (102 mg, 0.32 mmol) and PMe_3_ (36 mg, 0.48 mmol) was dissolved in THF (3 mL), stirred for 10 min at rt, and cooled to −37 °C. To this, a solution of compound **2** (0.32 mmol) in THF (2 mL) was added dropwise resulting in an orange–brown solution. After stirring for 1 h, the solvent was removed in a vacuum and the residue was extracted with pentane (3 × 2 mL). Filtration over celite was followed by concentration of the volume. Crystallization at −37 °C provided compound **9** (75 mg, 37%) as yellow–orange crystalline blocks. Mp.: 134–136 °C. NMR (δ in ppm): ^1^H: 1.13 (d, 9H, ^2^*J*_H-P_ = 9.9 Hz, PMe_3_), 0.58 (s, 12H, SiMe_2_), 0.44 (s, 18H, SiMe_3_), 0.39 (s, 18H, SiMe_3_). ^13^C: 18.6 (^1^*J*_C-P_ = 23 Hz, PMe_3_), 8.1 (OSiMe_2_), 4.6 (SiMe_3_), 3.1 (SiMe_3_). ^29^Si: 12.5 (d, ^3^*J*_Si-P_ = 16 Hz, OSiMe_2_), −9.2 (bs, SiMe_3_), −10.7 (bs, SiMe_3_), −134.3 (d, ^2^*J*_Si-P_ = 16 Hz, SiGe). ^31^P: −21.2.

#### 3.3.6. C-F Insertion Product from the Reaction of **9** with B(C_6_F_5_)_3_ (**10**)

To a solution of tris(pentafluorophenyl)borane (64 mg, 0.13 mmol) in pentane (4 mL), germylene adduct **9** in pentane (3 mL) was added dropwise. Immediately, the formation of a white precipitate in a clear and colorless solution was observed. After stirring for 30 min, the solvent was removed in a vacuum to yield a mixture of an oily residue and colorless crystalline blocks. The oily residue was removed by washing with cold pentane to leave compound **10** (87 mg, 64%). NMR (δ in ppm): ^1^H: 0.62 (d, 9H, ^2^*J*_P-H_ = 11.0 Hz, PMe_3_), 0.53 (s, 6H, SiMe_2_), 0.47 (s, 6H, SiMe_2_), 0.45 (s, 18H, SiMe_3_), 0.25 (s, 18H, SiMe_3_). ^13^C: 10.7 (bd, ^1^*J*_C-P_ = 40 Hz, PMe_3_), 7.7 (2 signals, SiMe_2_), 3.2 (SiMe_3_), 2.5 (SiMe_3_), signals for the fluorinated borate could not be detected. ^19^F: −127.5 (td, *J* = 16 Hz, 28 Hz, C_6_F_4_), −129.9 (pd, *J* = 16 Hz, C_6_F_4_), −130.0 (d, *J* = 26 Hz, o-C_6_F_5_), −155.8 (t, *J* = 23 Hz, p-C_6_F_5_), −163.3 (dt, *J* = 9 Hz, 24 Hz, m-C_6_F_5_), −209.2 (t, *J* = 17 Hz, Ge-F). ^29^Si: 11.4 (d, ^3^*J*_Si-F_ = 2.9 Hz, OSi), −6.5 (d, ^3^*J*_Si-F_ = 3.3 Hz, SiMe_3_), −8.6 (d, ^3^*J*_Si-F_ = 5.7 Hz, SiMe_3_), −109.9 (d, ^2^*J*_Si-F_ = 8.7 Hz, GeSi). ^31^P: −8.6 (bs, PMe_3_).

## 4. Conclusions

A number of compounds containing a 1,5-oligosiloxanylene unit were prepared. Repetitive formation of 1,5-oligosiloxanylene diides allowed the synthesis of a 3,7,10-trioxa-octasilabicyclo[3.3.3]undecane, with trimethylsilyl groups at the bridgehead positions. Reaction of 1,3-dichlorotetramethyldisiloxane with 1,1′-bis[bis(trimethylsilyl)potassiosilyl]ferrocene gave the respective ferrocenophane with a disiloxane containg bridge. The compound can be further derivatized by reaction with two equivalents of KO*^t^*Bu into the 1,5-oligosilanyl diide. NMR spectroscopic evidence for the presence of the latter was difficult. This was most likely due to fast configurational inversion of the silanide atoms; the dynamic of these processes leads to very broad resonances. Nevertheless, unequivocal proof for the dianion formation was obtained by derivatization with chlorosilanes.

Reacting 1,5-oligosiloxanylene diide **2** with SnCl_2_ or GeCl_2_·dioxane in the presence of PMe_3_ gave cyclic disilylated tetrylene PMe_3_ adducts. Spectroscopic and structural characteristics of these compounds are very similar to that of related compounds with 1,4-tetrasilanylene backbones.

Release of the base-free stannylene led to a dimerization process, which gave a distannene with two annulated seven membered rings. ^119^Sn NMR spectroscopic analysis showed the compound to be a distannene with a slight stannylene character also in solution featuring a resonance at 636.9 ppm. Abstraction of the PMe_3_ from the cyclic disilylated germylene PMe_3_ adduct with B(C_6_F_5_)_3_ was carried out to obtain a base-free disilylated germylene. Thus far, disilylated germylenes either dimerized to digermanes or underwent 1,2-silyl shift reactions to silagermenes, which either can dimerize in a [2 + 2] cycloaddition reaction or react further to a silylgermylsilylene, which inserts into a remote Si-Si bond. In the current case, however, oxidative addition of the germylene into a para-C-F bond of Me_3_P·B(C_6_F_5_)_3_ was observed. We assume that the novel reaction pattern is a consequence of a diminished singlet-triplet gap of the germylene, which is caused by the geometrical constraint of the elongated backbone resulting in a larger Si-Ge-Si angle and thus an increased triplet character.

## Supplementary Materials

The supplementary materials are available online. Figure S1–S22, ^1^H, ^13^C, ^19^F, ^29^Si, ^31^P, and ^119^Sn NMR spectra of compounds **5**, **5****a**, **7**, **8**, **9**, and **10**, Table S1, Tabulated crystallographic data and ORTEP plots for compounds **3**, **5**, **7**, **8**, **9**, and **10**.
