# Supplementary material for: A 1,5-Oligosilanylene Dianion as Building Block for Oligosiloxane Containing Cages, Ferrocenophanes, and Cyclic Germylenes and Stannylenes
Source: Molecules. 2020 Mar 13;25(6):1322. doi: 10.3390/molecules25061322 (PMC7144727; doi:10.3390/molecules25061322)
Supplement: Supplementary file 1 [file molecules-25-01322-s001.pdf]

molecules

supplementary materials

A 1,5-Oligosilanylene Dianion as Building Block for Oligosiloxane Containing Cages, Ferrocenophanes, and Cyclic Germylenes and Stannylenes

Rainer Zitz, Alexander Pöcheim, Judith Baumgartner * and Christoph Marschner *

Institut für Anorganische Chemie, Technische Universität Graz, Stremayrgasse 9, A-8010 Graz, Austria; Rainer_Zitz@hotmail.com (R.Z.); alexander.poecheim@tugraz.at (A.P.).

* Correspondence: christoph.marschner@tugraz.at (C.M.); baumgartner@tugraz.at (J.B.); Tel.: +43-316-873-32112 (C.M.); +43-316-873-32107 (J.B.)

Table 1. Crystallographic data for compounds **3**, **5**, **7**, **8**, **9**, and **10**

	**3**	**5**	**7**	**8**	**9**	**10**
Empirical formula	$C_{18}H_{54}O_3Si_{10}$	$C_{26}H_{56}FeOSi_8$	$C_{19}H_{57}OPSi_8Sn$	$C_{32}H_{96}O_2Si_{16}Sn_2$	$C_{19}H_{57}GeOPSi_8$	$C_{37}H_{57}BF_{15}GeOPSi_8$
M_w	599.48	665.28	676.03	1199.91	629.93	1141.92
Temperature [K]	100(2)	100(2)	100(2)	100(2)	100(2)	100(2)
Size [mm]	0.26×0.19×0.12	0.34×0.25×0.20	0.22×0.20×0.16	0.48×0.32×0.30	0.30×0.24×0.14	0.32×0.27×0.12
Crystal system	tetragonal	orthorhombic	orthorhombic	monoclinic	monoclinic	monoclinic
Space group	P4(1)	C222(1)	Pbcn	C2/c	P2(1)	P2(1)/c
a [Å]	11.487(2)	14.236(3)	21.589(5)	27.786(8)	9.517(2)	12.313(2)
b [Å]	11.487(2)	17.083(3)	15.740(4)	12.559(3)	16.994(2)	24.660(4)
c [Å]	28.192(6)	15.739(3)	22.217(5)	19.480(5)	11.952(4)	19.855(3)
α [°]	90	90	90	90	90	90
β [°]	90	90	90	109.766(6)	110.057(4)	116.210(8)
γ [°]	90	90	90	90	90	90
V [Å ^{3}]	3720(2)	3828(2)	7550(3)	6397(3)	1816(2)	5409(2)
Z	4	4	8	4	2	4
ρ_{calc} [gcm^{-3}]	1.069	1.154	1.190	1.246	1.152	1.402
Absorption coefficient [mm^{-1}]	0.370	0.662	0.984	1.105	1.163	0.854
F(000)	1300	1432	2848	2512	676	2344
θ range	1.77<θ<26.37	1.86<θ<26.34	1.60<θ<26.37	1.80<θ<26.34	1.81<θ<26.37	1.41<θ<26.36
Reflections collected/unique	25491/7548	15151/3891	57304/7718	20761/6396	14473/7243	42766/11016
Completeness to θ [%]	99.8	99.9	99.9	98.1	99.5	99.5
Data/restraints/parameters	7548/1/297	3891/0/173	7718/0/290	6396/0/252	7243/1/291	11016/0/596
Goodness of fit on F^2	1.04	1.43	1.09	1.29	1.03	1.21
Final R indices [I>2σ(I)]	R1=0.047, *w*R2=0.123	R1=0.037, *w*R2=0.085	R1=0.035, *w*R2=0.079	R1=0.030, *w*R2=0.067	R1=0.048, *w*R2=0.111	R1=0.074, *w*R2=0.146
R indices (all data)	R1=0.047, *w*R2=0.120	R1=0.038, *w*R2=0.085	R1=0.040, *w*R2=0.081	R1=0.033, *w*R2=0.067	R1=0.052, *w*R2=0.112	R1=0.091, *w*R2=0.152
Largest diff. Peak/hole [e$^-$/ Å^3]	0.43/-0.77	0.38/-0.24	0.85/-0.30	1.12/-0.43	1.56/-0.77	1.16/-0.49

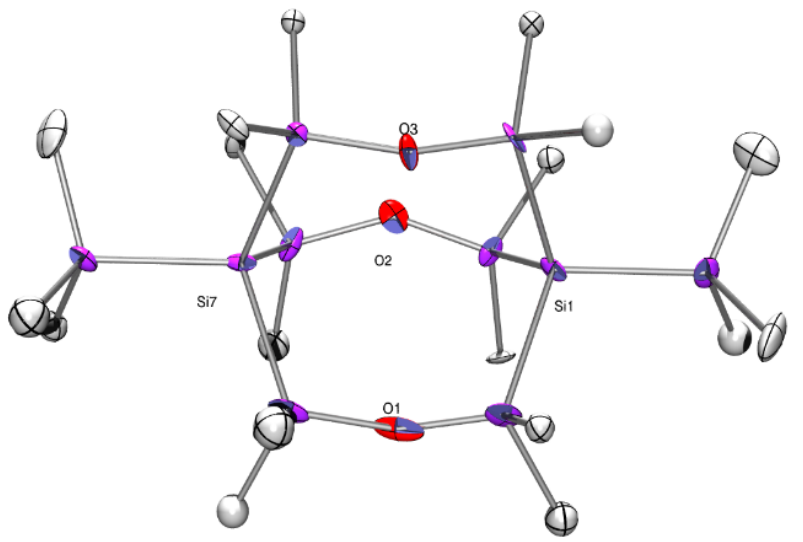

Figure 1. Molecular structure of **3** (thermal ellipsoid plot drawn at the 30% probability level). All hydrogen atoms are omitted for clarity (bond lengths in Å, angles in deg). Si(1)-Si(4) 2.3404(18), Si(1)-Si(3) 2.3430(18), Si(1)-Si(2) 2.3486(18), Si(1)-Si(5) 2.3497(18), Si(4)-O(3) 1.641(4), Si(6)-O(3) 1.647(4), Si(8)-O(1) 1.648(4), Si(4)-Si(1)-Si(3) 108.90(7), Si(4)-Si(1)-Si(2) 110.01(7), Si(3)-Si(1)-Si(2) 110.54(7), Si(4)-Si(1)-Si(5) 110.13(7), Si(3)-Si(1)-Si(5) 107.46(7), Si(2)-Si(1)-Si(5) 109.76(7), Si(10)-O(2)-Si(3) 147.9(2), Si(4)-O(3)-Si(6) 148.2(2).

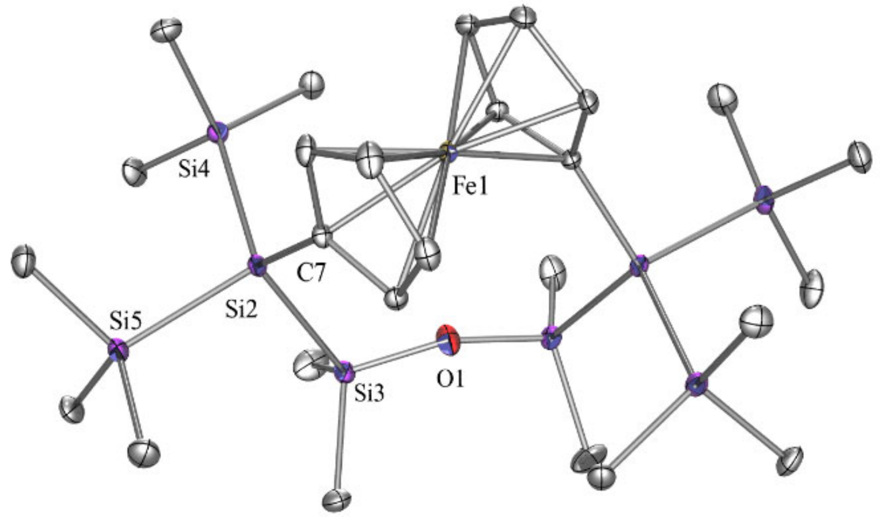

Figure 2. Molecular structure of **5** (thermal ellipsoid plot drawn at the 30% probability level). All hydrogen atoms are omitted for clarity (bond lengths in Å, angles in deg). Fe(1)-C(7) 2.080(3), O(1)-Si(3) 1.6250(11), Si(2)-C(7) 1.880(3), Si(2)-Si(3) 2.3474(12), Si(2)-Si(4) 2.3507(12), Si(2)-Si(5) 2.3555(12), C(7)-Si(2)-Si(3) 108.30(10), C(7)-Si(2)-Si(4) 114.63(10),Si(3)-Si(2)-Si(4) 111.57(4), Si(2)-C(7)-Fe(1) 131.76(15).

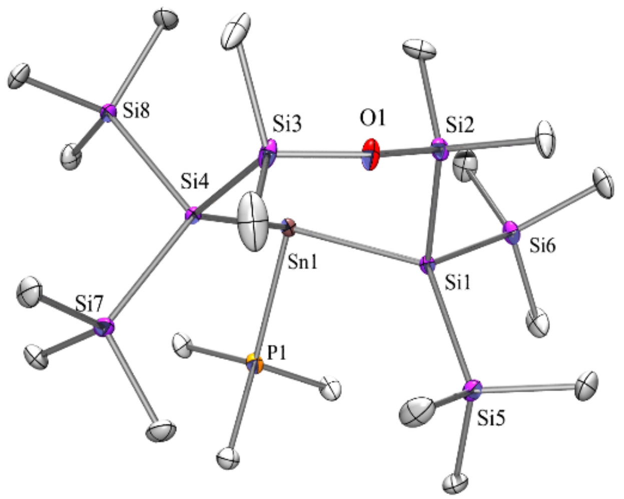

Figure 3. Molecular structure of **7** (thermal ellipsoid plot drawn at the 30% probability level). All hydrogen atoms are omitted for clarity (bond lengths in Å, angles in deg). C(1)-P(1) 1.814(3), C(4)-Si(2) 1.866(3), O(1)-Si(3) 1.6378(19), O(1)-Si(2) 1.642(2), P(1)-Sn(1) 2.6059(8), Si(1)-Si(2) 2.3397(11), Si(1)-Sn(1) 2.6464(8), Si(4)-Sn(1) 2.6662(8), Si(3)-O(1)-Si(2) 145.51(13), Si(2)-Si(1)-Sn(1) 96.56(3), Si(3)-Si(4)-Sn(1) 109.20(3), P(1)-Sn(1)-Si(1) 99.58(2), P(1)-Sn(1)-Si(4) 104.33(2), Si(1)-Sn(1)-Si(4) 103.35(2).

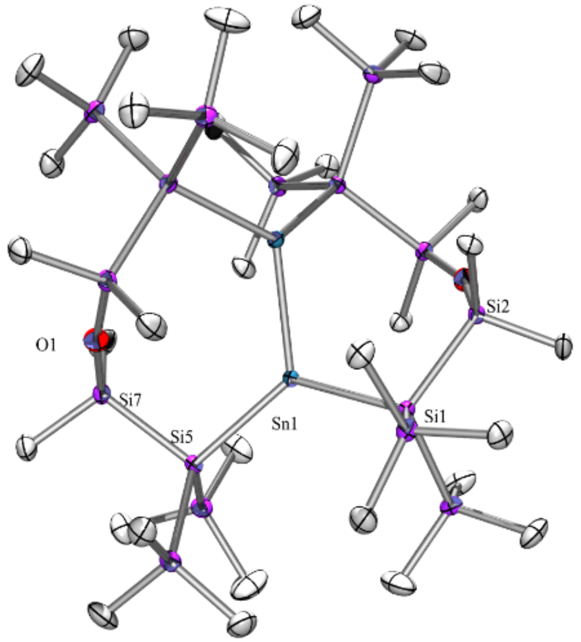

Figure 4. Molecular structure of **8** (thermal ellipsoid plot drawn at the 30% probability level). All hydrogen atoms are omitted for clarity (bond lengths in Å, angles in deg). Sn(1)-Si(1) 2.6131(8), Sn(1)-Si(5) 2.6179(8), Sn(1)-Sn(1A) 2.7409(9), Si(1)-Si(2) 2.3557(10), Si(7)-O(1) 1.6302(8), Si(7)-C(14) 1.866(3), Si(1)-Sn(1)-Si(5) 122.90(3), Si(1)-Sn(1)-Sn(1A) 105.336(17), Si(5)-Sn(1)-Sn(1A) 122.683(18), Si(7)-O(1)-Si(7A) 168.10(19).

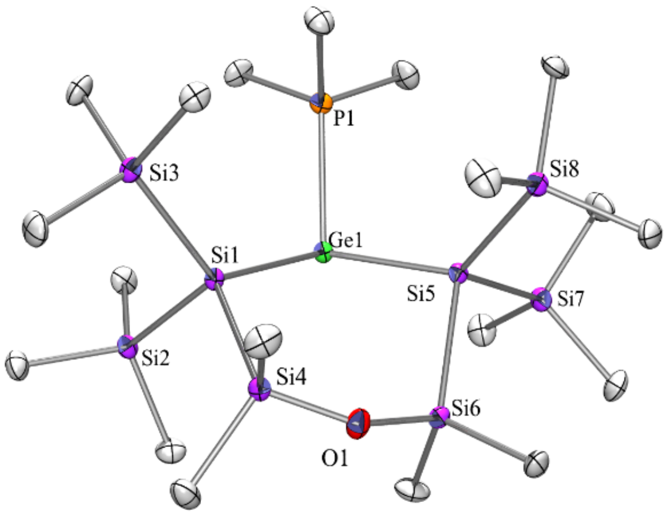

Figure 5. Molecular structure of **9** (thermal ellipsoid plot drawn at the 30% probability level). All hydrogen atoms are omitted for clarity (bond lengths in Å, angles in deg). Ge(1)-P(1) 2.3570(13), Ge(1)-Si(5) 2.4463(13), Ge(1)-Si(1) 2.4664(13), O(1)-Si(4) 1.625(3), O(1)-Si(6) 1.642(3), P(1)-C(1) 1.818(5), Si(1)-Si(4) 2.3656(17), Si(2)-C(9) 1.879(5), Si(5)-Si(6) 2.3537(17), P(1)-Ge(1)-Si(5) 100.20(5), P(1)-Ge(1)-Si(1) 101.87(4), Si(5)-Ge(1)-Si(1) 107.88(5), Si(4)-O(1)-Si(6) 148.4(2), Si(4)-Si(1)-Ge(1) 110.79(6).

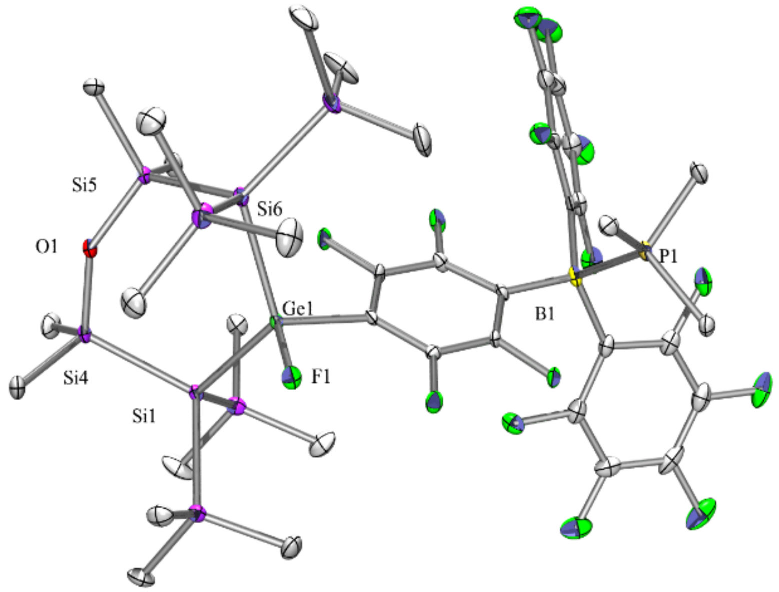

Figure 6. Molecular structure of **10** (thermal ellipsoid plot drawn at the 30% probability level). All hydrogen atoms are omitted for clarity (bond lengths in Å, angles in deg). Ge(1)-F(1) 1.769(2), Ge(1)-C(17) 1.969(4), Ge(1)-Si(6) 2.3849(13), Ge(1)-Si(1) 2.3966(12), O(1)-Si(5) 1.654(3), O(1)-Si(4) 1.654(3), P(1)-C(35) 1.805(5), P(1)-B(1) 2.055(5), Si(1)-Si(4) 2.3775(17), Si(2)-C(1) 1.860(5), Si(5)-Si(6) 2.3447(17), B(1)-C(29) 1.632(7), F(10)-C(28) 1.347(6), F(1)-Ge(1)-Si(6) 105.08(9), F(1)-Ge(1)-Si(1) 104.92(9), Si(6)-Ge(1)-Si(1) 119.41(4), Si(5)-O(1)-Si(4) 139.1(2), Si(4)-Si(1)-Ge(1) 107.01(6), Si(5)-Si(6)-Ge(1) 101.08(5).

NMR Spectra

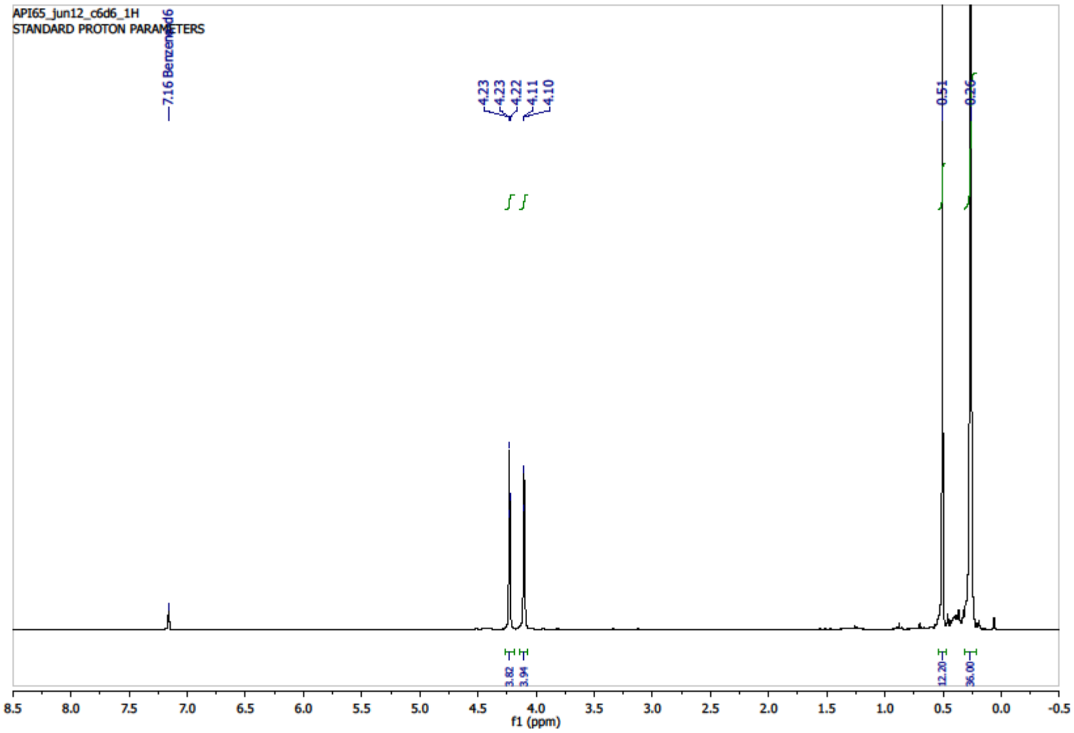

Figure S1. ^{1}H NMR spectrum of **5** in C_6D_6.

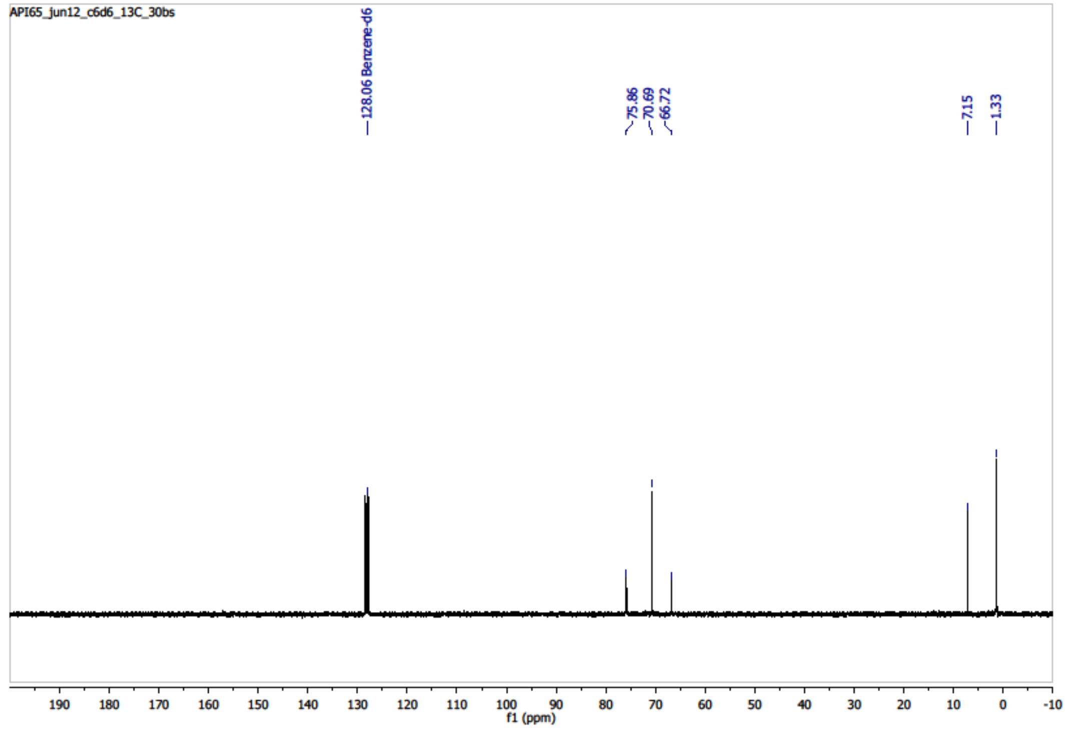

Figure S2. ^{13}C{H} NMR spectrum of **5** in C_6D_6.

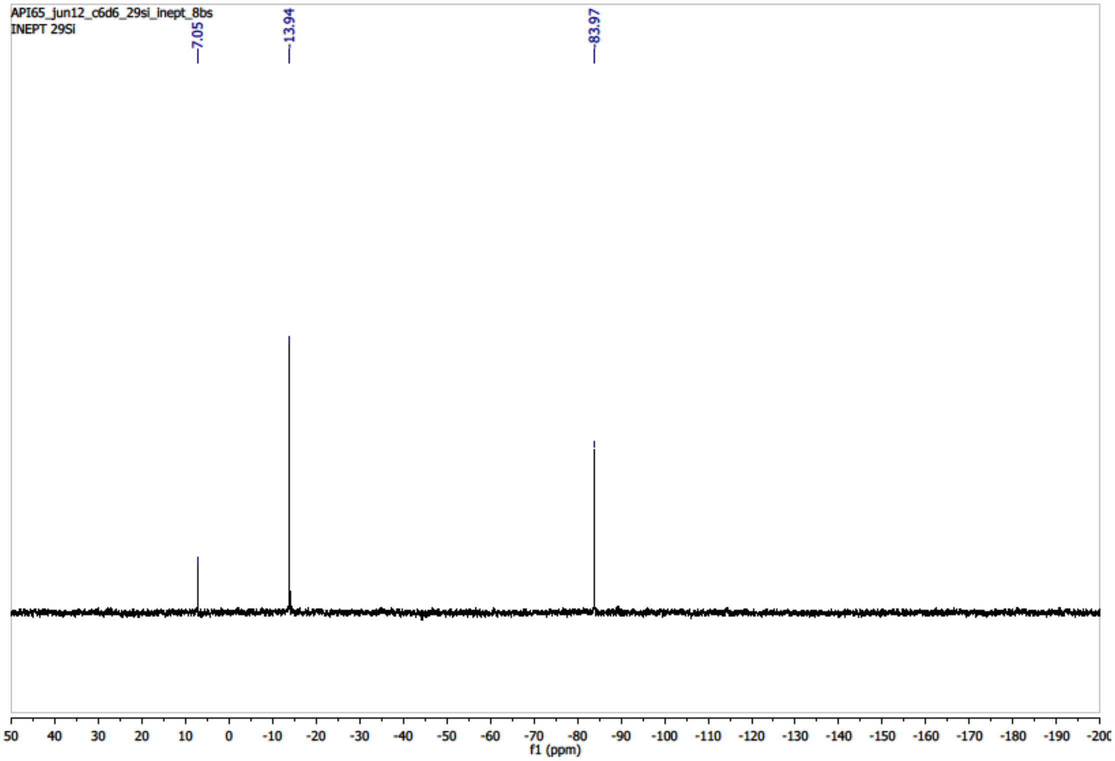

Figure S3. $^{29}Si\{H\}$ INEPT NMR spectrum of **5** in C_6D_6.

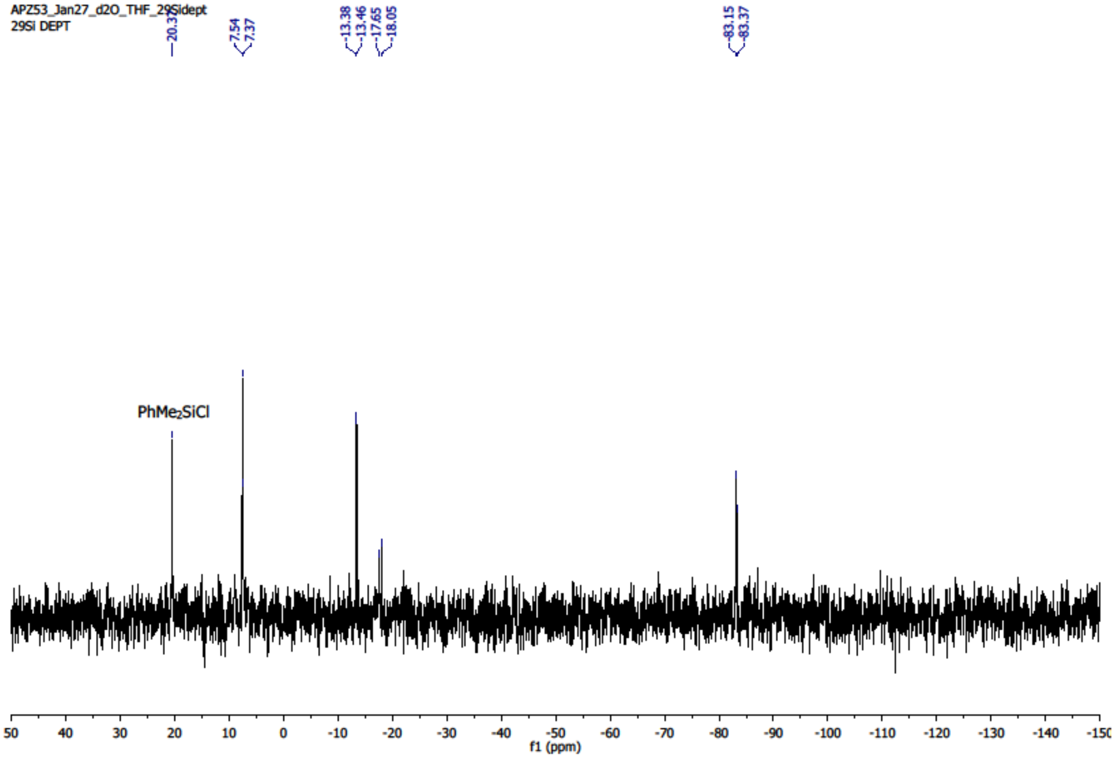

Figure S4. $^{29}Si\{H\}$ DEPT NMR spectrum of **5a** in THF (with excess $PhMe_2SiCl$ present).

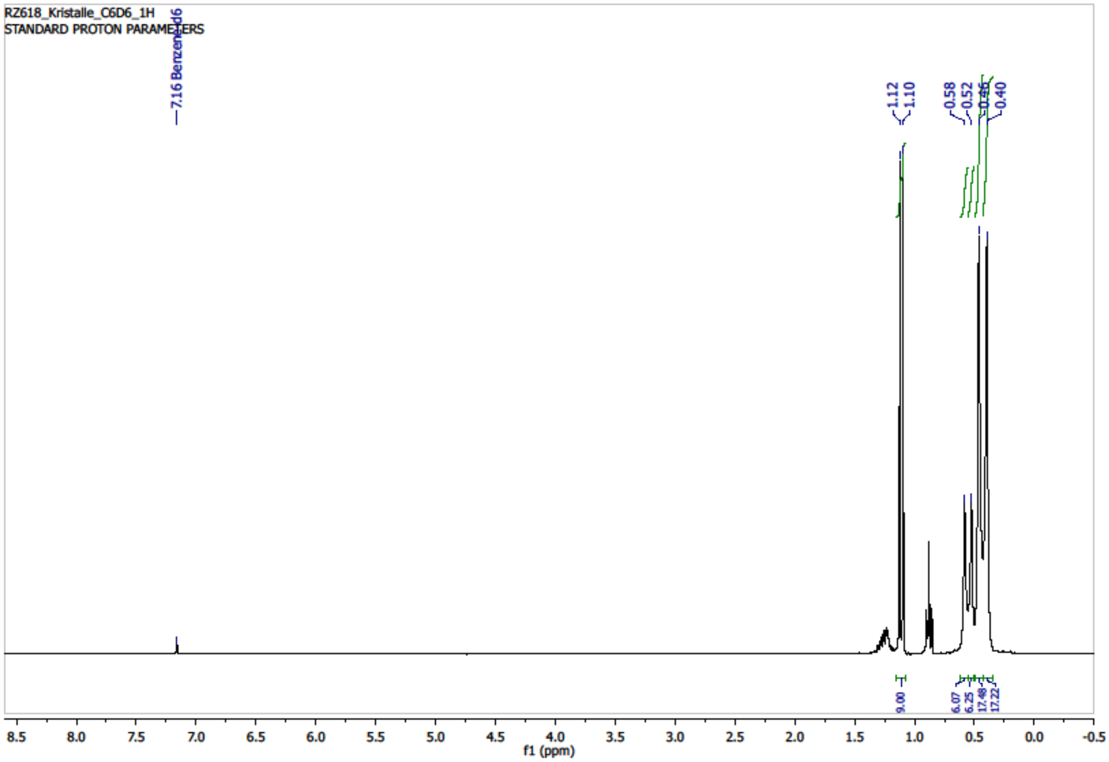

Figure S5. ^{1}H NMR spectrum of **7** in C_6D_6 (containing co-crystallized pentane).

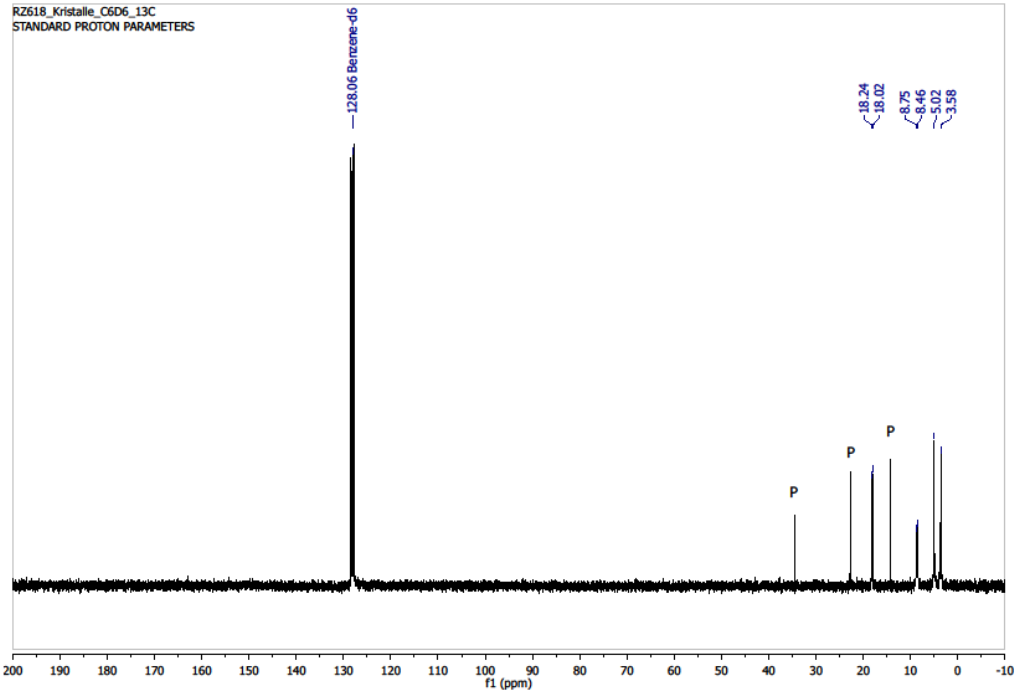

Figure S6. $^{13}C\{H\}$ NMR spectrum of **7** in C_6D_6 (P denotes co-crystallized pentane).

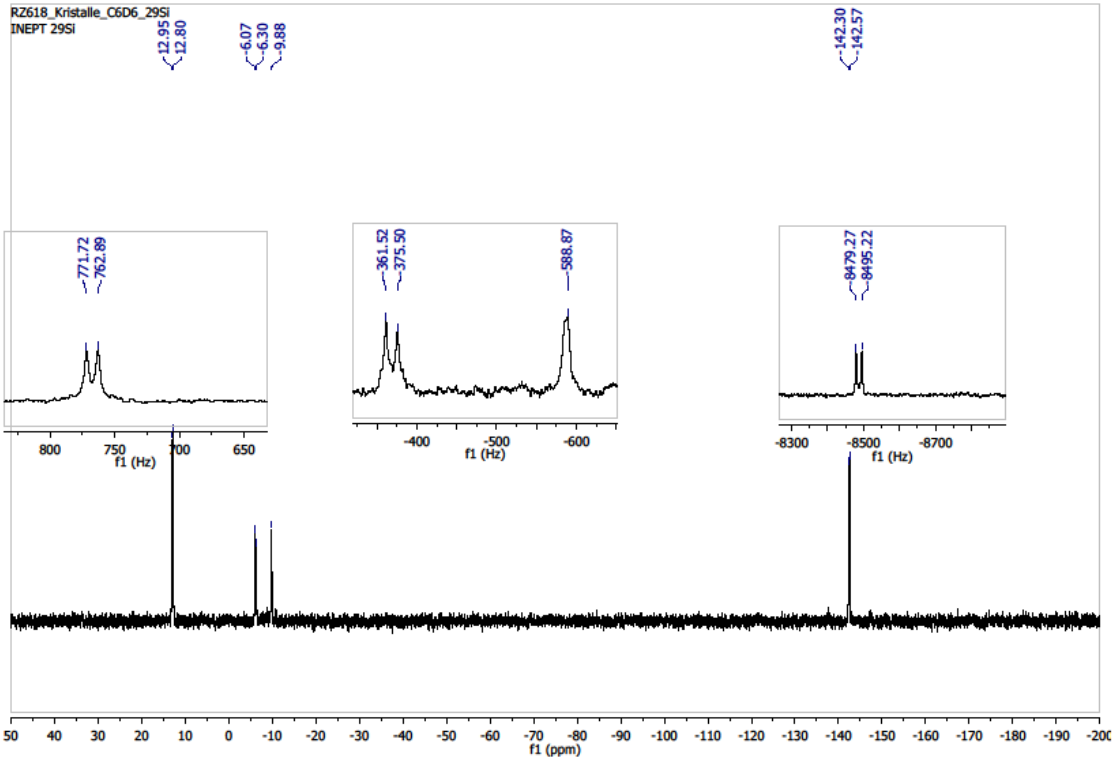

Figure S7. $^{29}Si\{H\}$ INEPT NMR spectrum of **7** in C_6D_6.

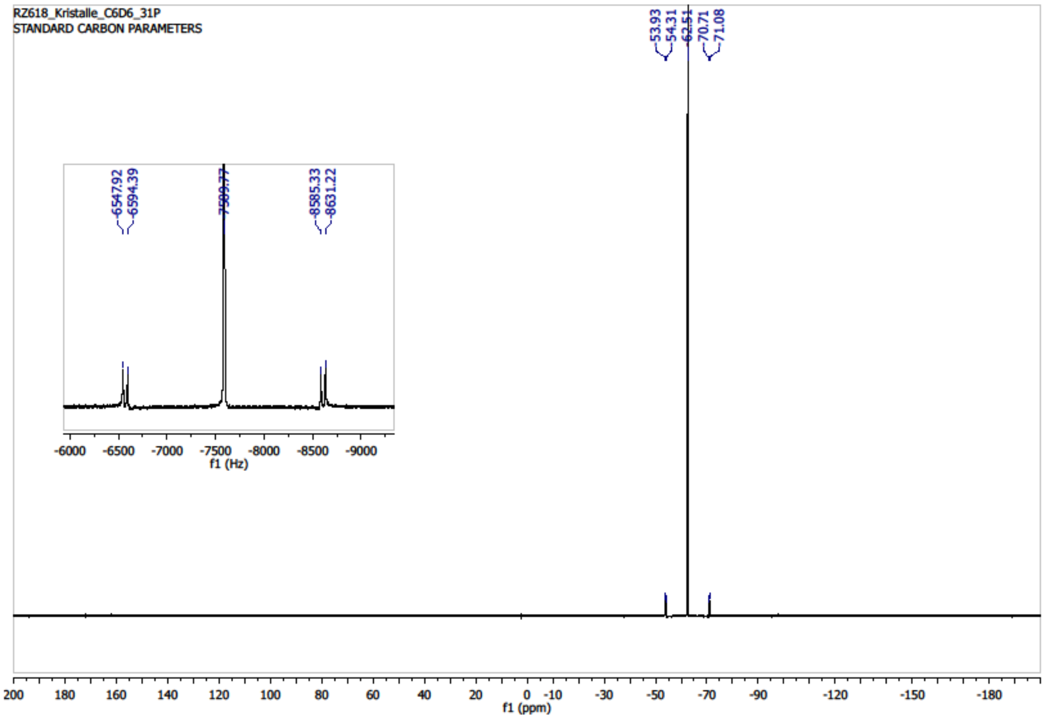

Figure S8. $^{31}P\{H\}$ NMR spectrum of **7** in C_6D_6.

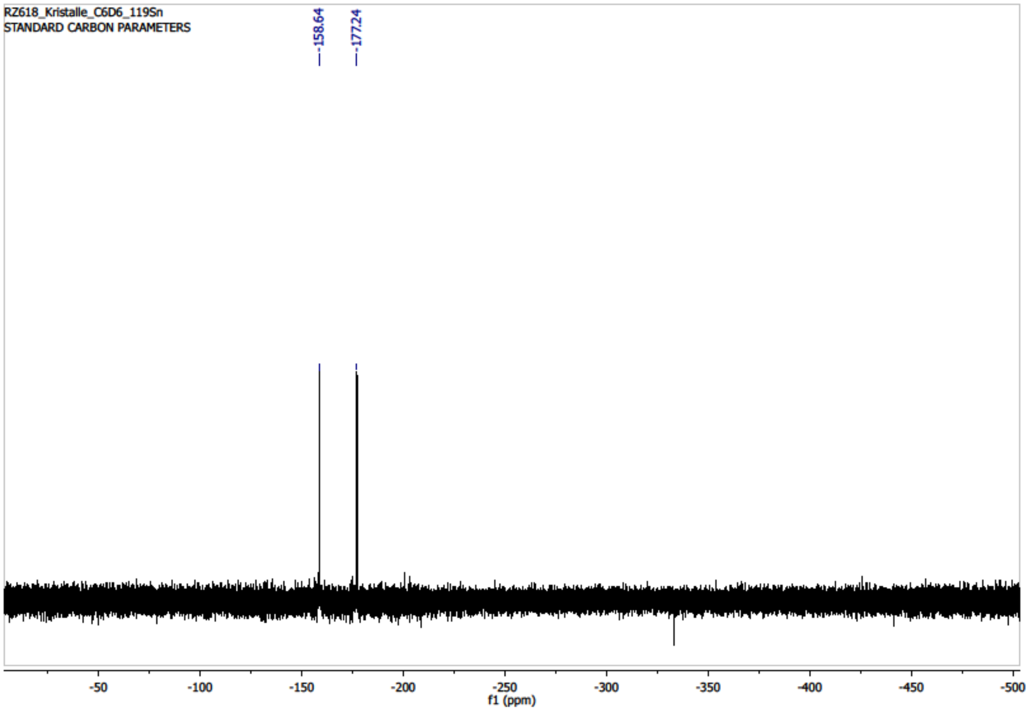

Figure S9. $^{119}Sn\{H\}$ NMR spectrum of **7** in C_6D_6.

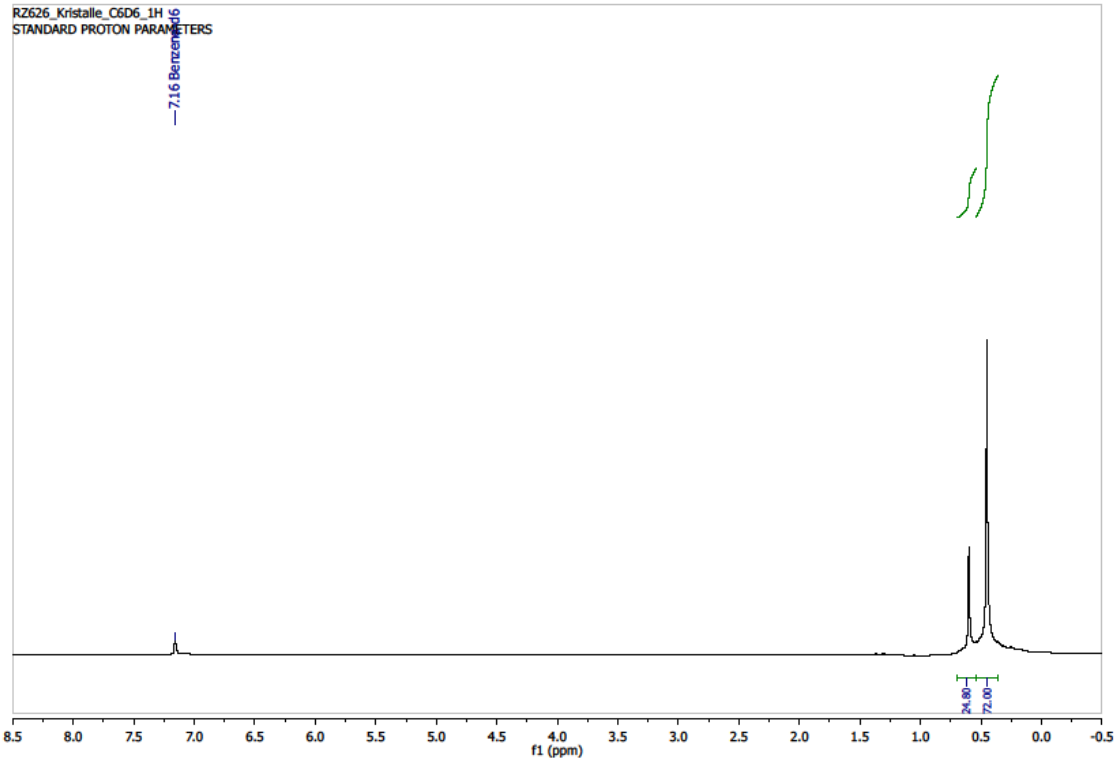

Figure S10. ^{1}H NMR spectrum of **8** in C_6D_6.

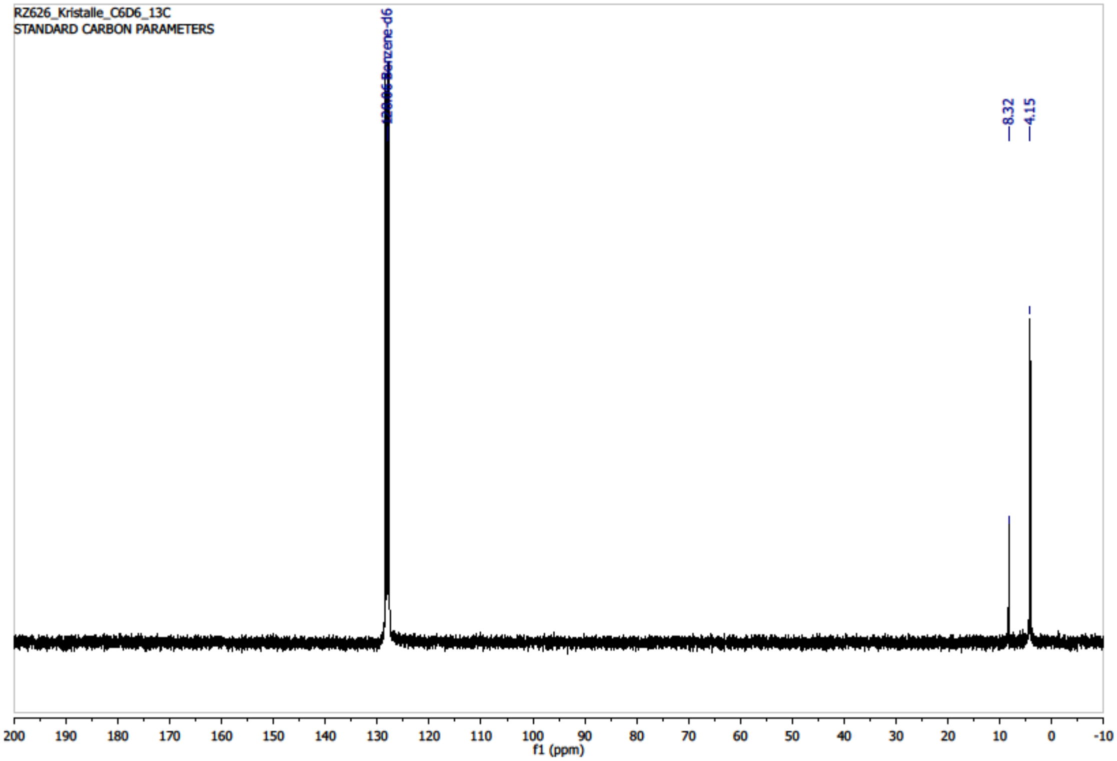

Figure S11. $^{13}C\{H\}$ NMR spectrum of **8** in C_6D_6.

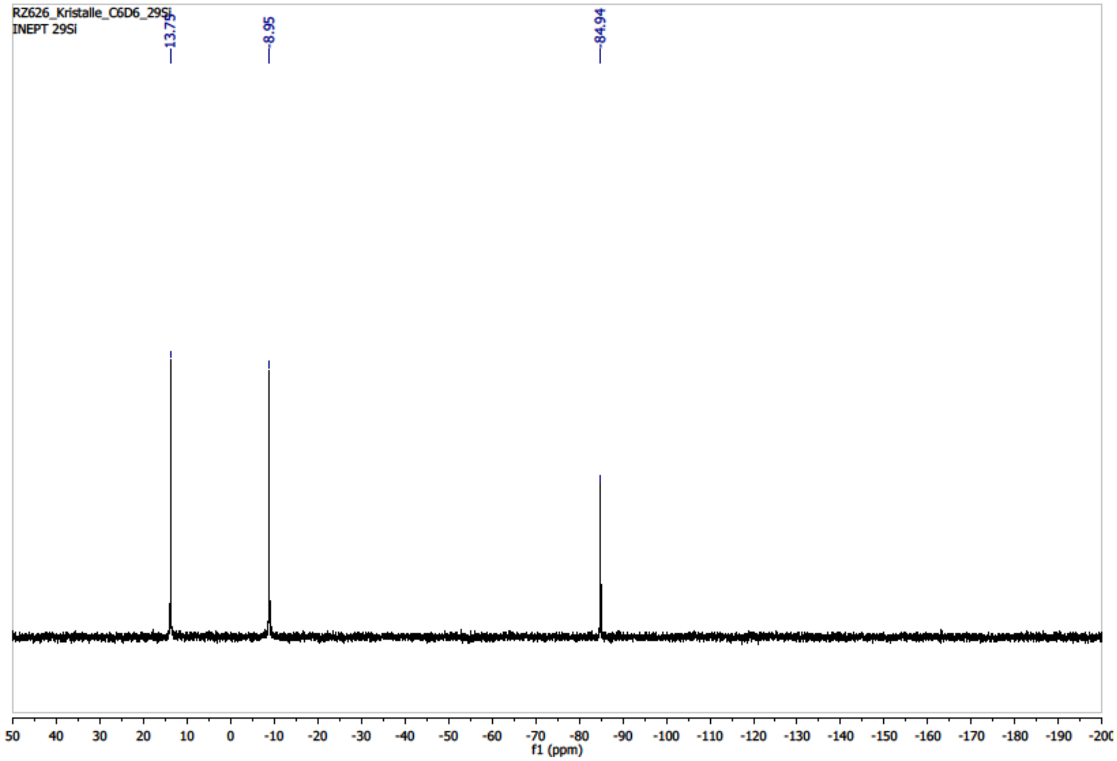

Figure S12. $^{29}Si\{H\}$ INEPT NMR spectrum of **8** in C_6D_6.

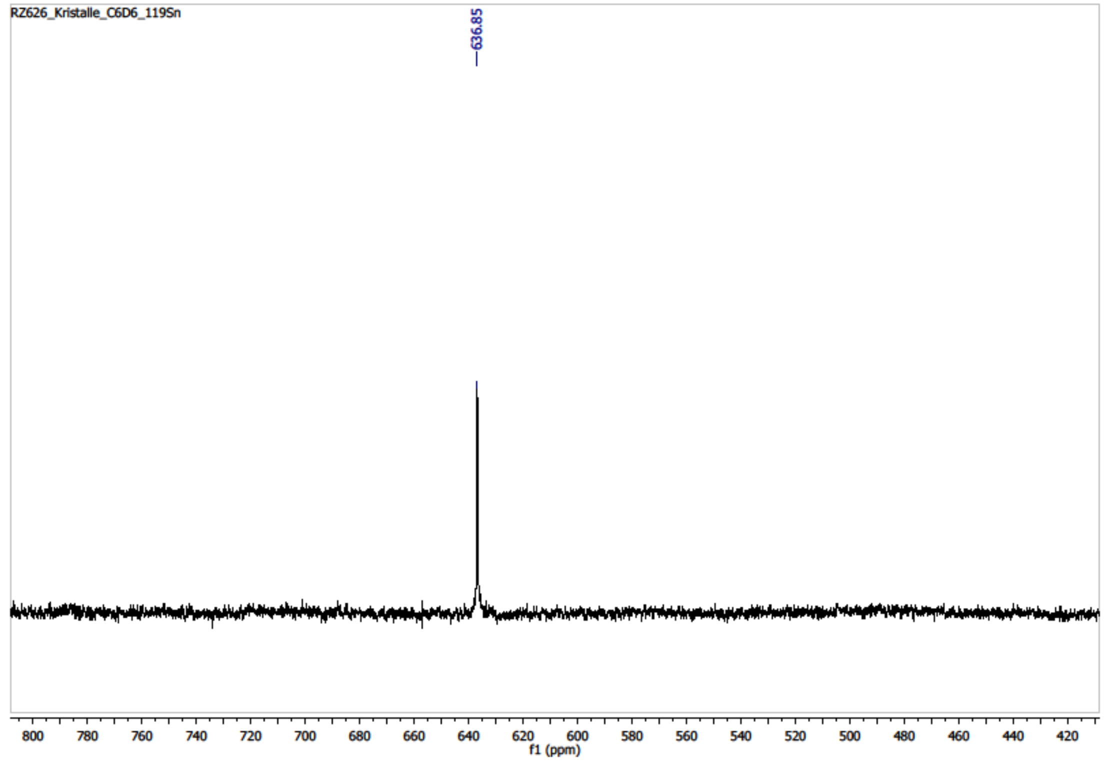

Figure S13. $^{119}Sn\{H\}$ NMR spectrum of **8** in C_6D_6.

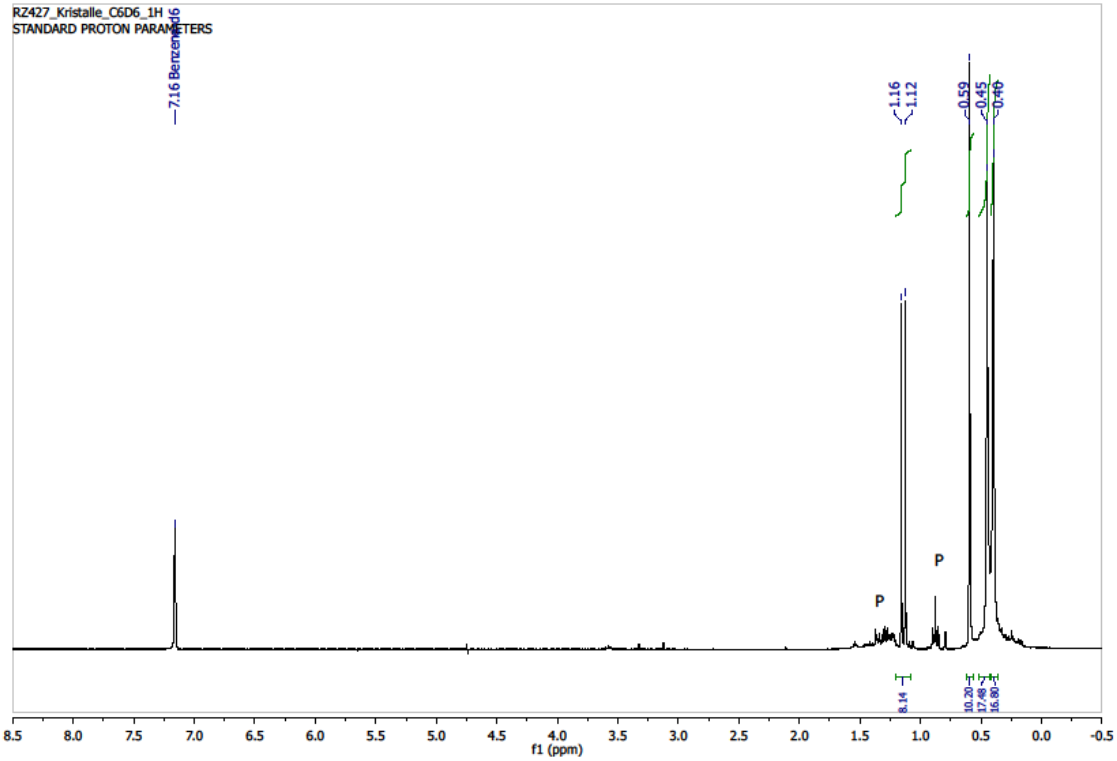

Figure S14. ^{1}H NMR spectrum of **9** in C_6D_6 (P denotes co-crystallized pentane).

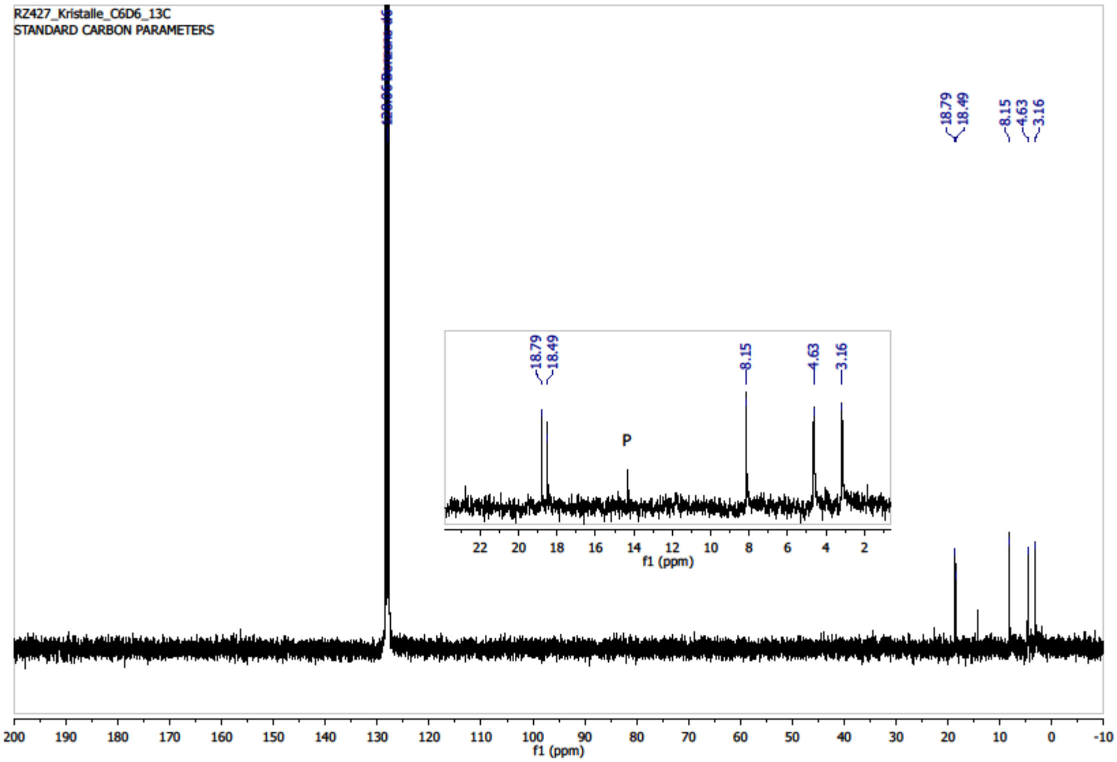

Figure S15. $^{13}C\{H\}$ NMR spectrum of **9** in C_6D_6 (P denotes co-crystallized pentane).

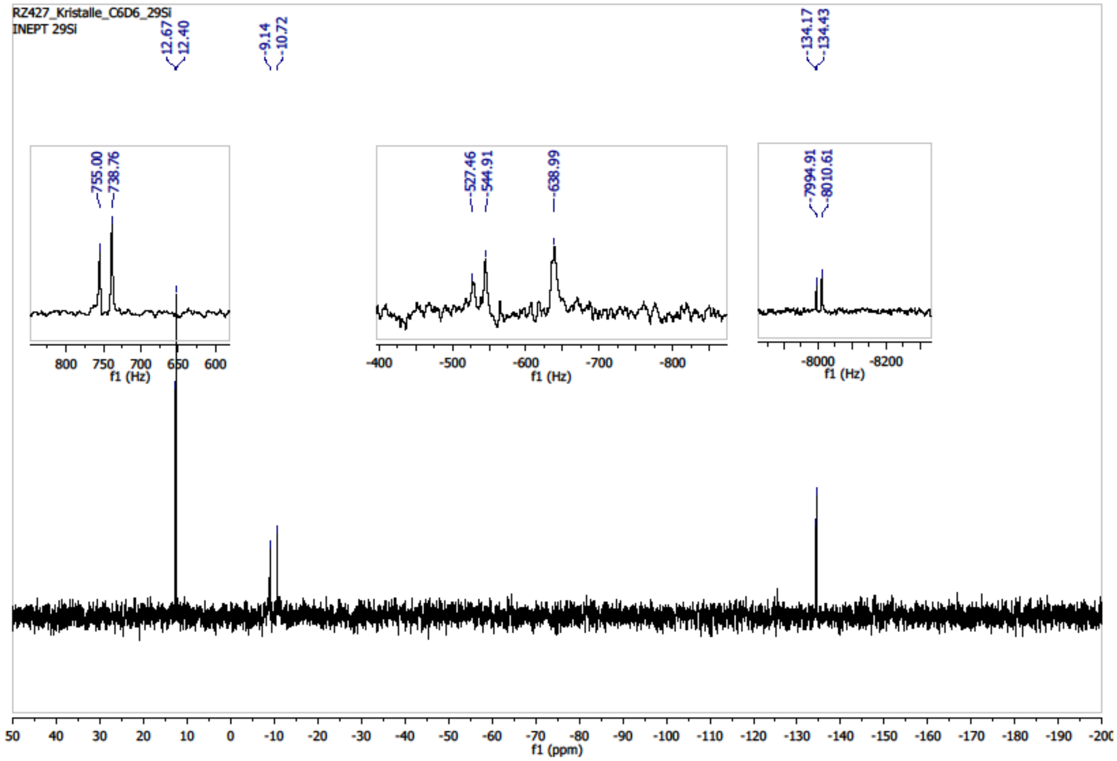

Figure S16. $^{29}Si\{H\}$ INEPT NMR spectrum of **9** in C_6D_6.

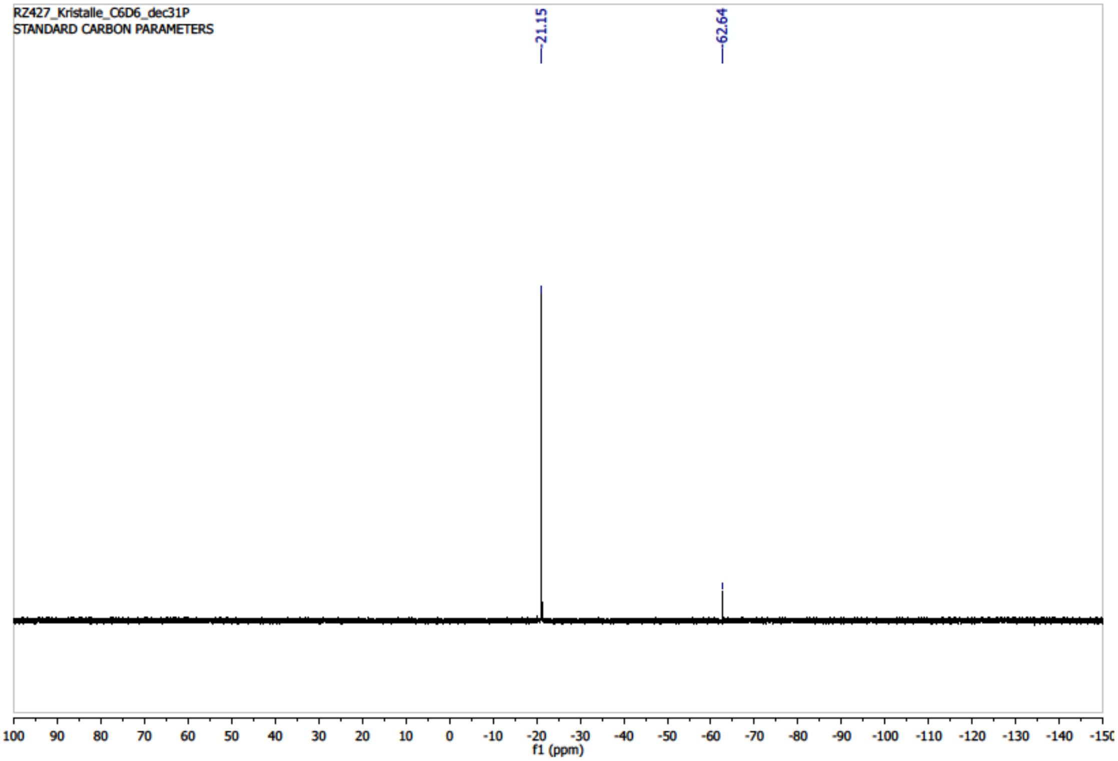

Figure S17. $^{31}P\{H\}$ NMR spectrum of **9** in C_6D_6 (signal at -62.6 is free PMe_3).

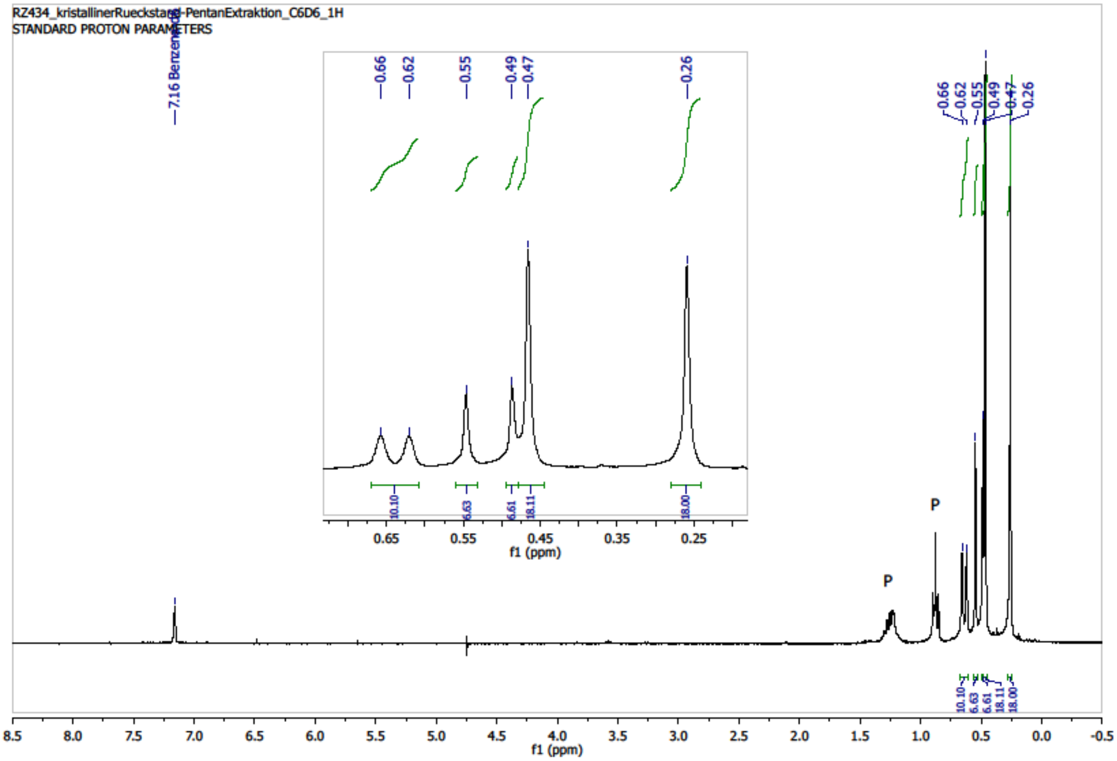

Figure S18. ^{1}H NMR spectrum of **10** in C_6D_6.

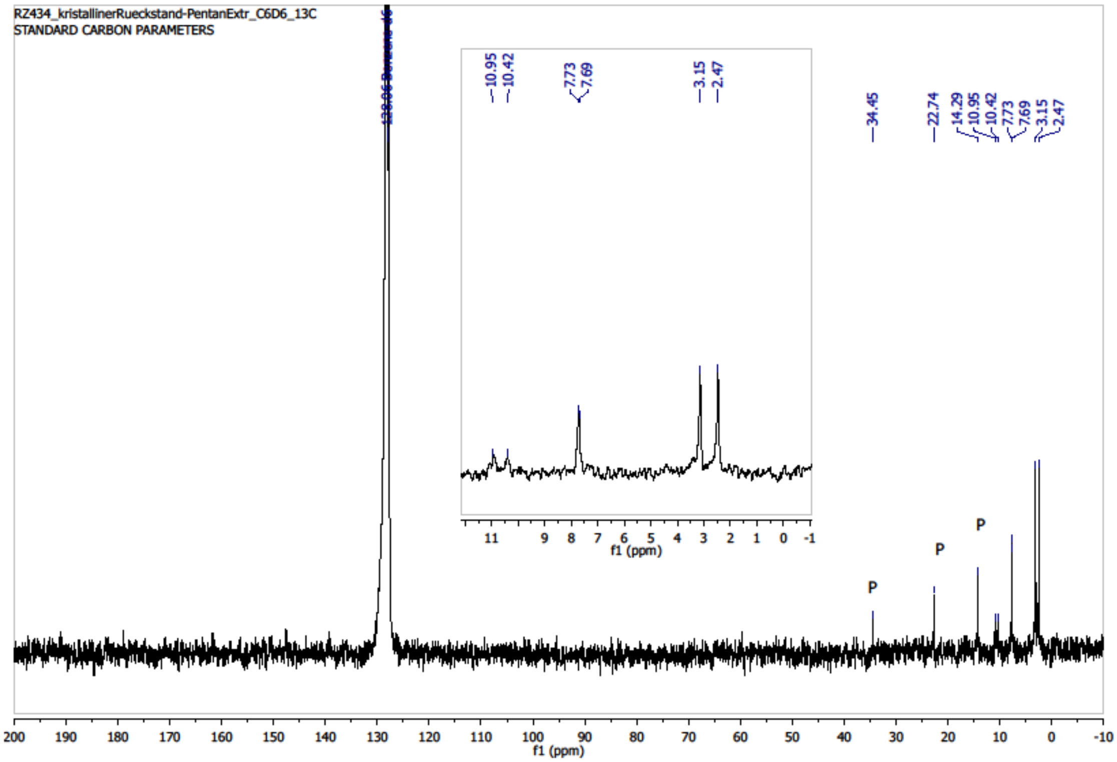

Figure S19. $^{13}C\{H\}$ NMR spectrum of **10** in C_6D_6.

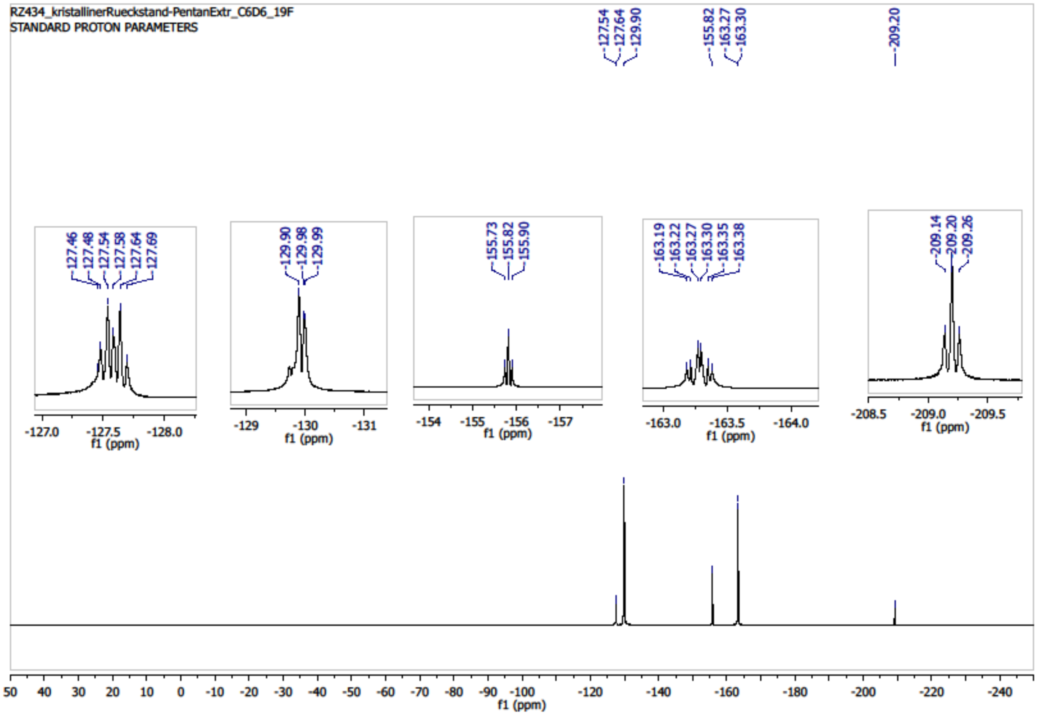

Figure S20. $^{19}F\{H\}$ NMR spectrum of **10** in C_6D_6.

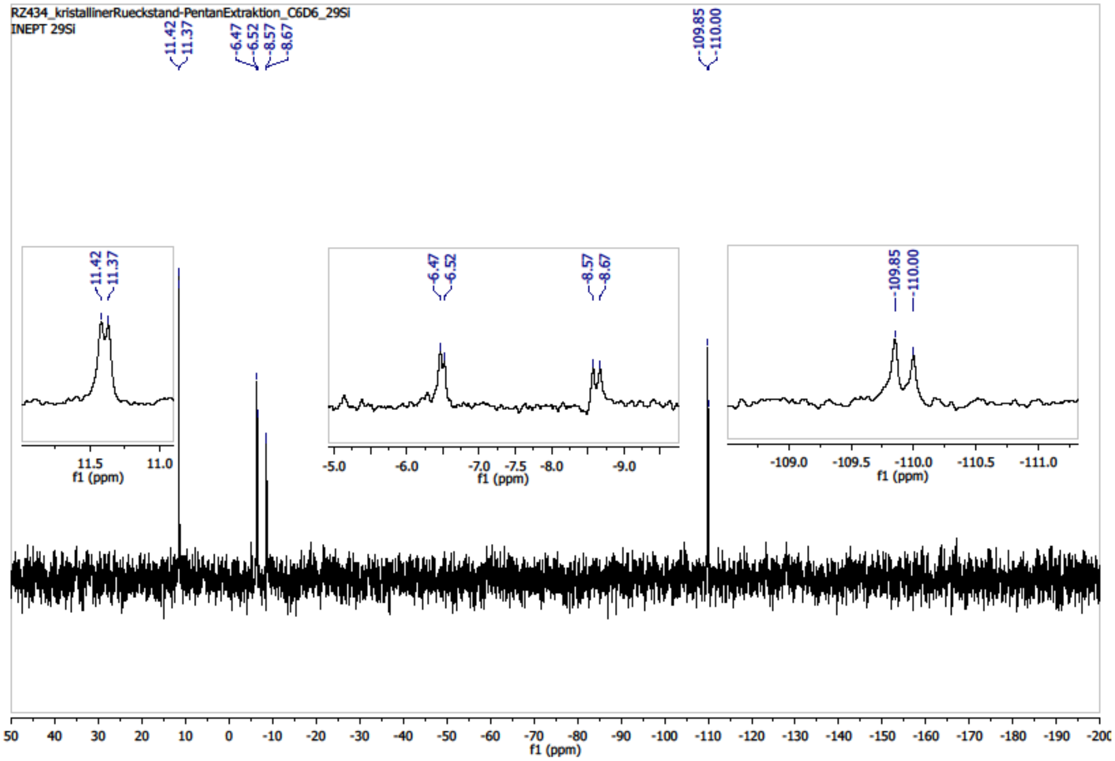

Figure S21. $^{29}Si\{H\}$ INEPT NMR spectrum of **10** in C_6D_6.

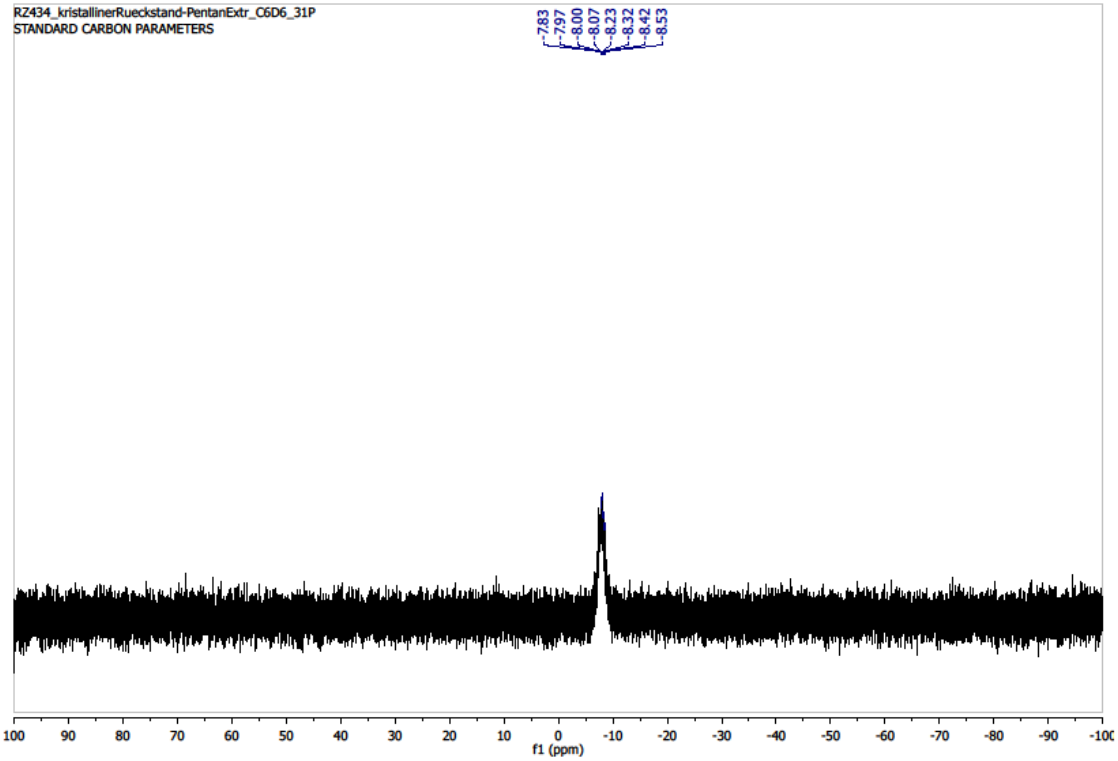

Figure S22. $^{31}P\{H\}$ NMR spectrum of **10** in C_6D_6.
